# Synthesizing adaptive test strategies from temporal logic specifications

**DOI:** 10.1007/s10703-019-00338-9

**Published:** 2019-10-14

**Authors:** Roderick Bloem, Goerschwin Fey, Fabian Greif, Robert Könighofer, Ingo Pill, Heinz Riener, Franz Röck

**Affiliations:** 1 Graz University of Technology, Graz, Austria; 2 Hamburg University of Technology, Hamburg, Germany; 3 German Aerospace Center, Bremen, Germany; 4 EPFL, Lausanne, Switzerland

**Keywords:** Automatic test case generation, System testing, Specification testing, Adaptive tests, Synthesis, Reactive systems, Mutation testing

## Abstract

Constructing good test cases is difficult and time-consuming, especially if the system under test is still under development and its exact behavior is not yet fixed. We propose a new approach to compute test strategies for reactive systems from a given temporal logic specification using formal methods. The computed strategies are guaranteed to reveal certain simple faults in *every* realization of the specification and for *every* behavior of the uncontrollable part of the system’s environment. The proposed approach supports different assumptions on occurrences of faults (ranging from a single transient fault to a persistent fault) and by default aims at unveiling the weakest one. We argue that such tests are also sensitive for more complex bugs. Since the specification may not define the system behavior completely, we use reactive synthesis algorithms with partial information. The computed strategies are *adaptive test strategies* that react to behavior at runtime. We work out the underlying theory of adaptive test strategy synthesis and present experiments for a safety-critical component of a real-world satellite system. We demonstrate that our approach can be applied to industrial specifications and that the synthesized test strategies are capable of detecting bugs that are hard to detect with random testing.

## Introduction

Model checking [12, 48] is an algorithmic approach to prove that a model of a system adheres to its specification. However, model checking cannot always be applied effectively to obtain confidence in the correctness of a system. Possible reasons include scalability issues, third-party IP components for which no code or detailed model is available, or a high effort for building system models that are sufficiently precise. Moreover, model checking cannot verify the final and “live” product but only an (abstracted) model.

Testing is a natural alternative to complement formal methods like model checking, and automatic test case generation helps keeping the effort acceptable. Black-box testing techniques, where tests are derived from a specification rather than the implementation, are particularly attractive: first, tests can be computed before the implementation phase starts, and thus guide the development. Second, the same tests can be reused across different realizations of a given specification. Third, a specification is usually much simpler than its implementation, which gives a scalability advantage. At the same time, the specification focuses on critical functional aspects that require thorough testing. Fault-based techniques [29] are particularly appealing, where the computed tests are guaranteed to reveal all faults in a certain fault class—after all, the foremost goal in testing is to detect bugs.

Methods to derive tests from declarative requirements (see, e.g., [25]) are sparse. One issue in this setting is controllability: the requirements leave plenty of implementation freedom, so they cannot be used to fully predict the system behavior for all given inputs. Consequently, test cases have to be *adaptive*, i.e., able to react to observed behavior at runtime, rather than being fixed input sequences. This is particularly true for *reactive systems* that continuously interact with their environment. Existing methods often work around this complication by requiring a deterministic system model as additional input [24]. Even a probabilistic model fixes the behavior in a way not necessarily required by the specification.

In previous work, we presented a fault-based approach to compute adaptive test strategies for reactive systems [10]. This approach generates tests that enforce certain coverage goals for *every* implementation of a provided specification. The generated tests can be used across realizations of the specification that differ not only in implementation details but also in their observable behavior. This is, e.g., useful for standards and protocols that are implemented by multiple vendors or for systems under development, where the exact behavior is not yet fixed.

**Fig. 1:**
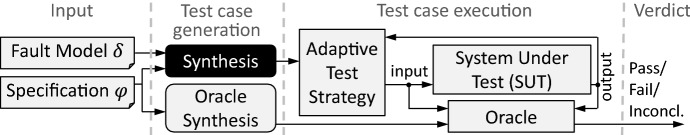
Testing setup: this paper focuses on test strategy synthesis

Figure 1 outlines the assumed testing setup and shows how the approach for synthesizing adaptive test strategies (illustrated in black) can be integrated in an existing testing flow.

The user provides a specification $\varphi$, which describes the requirements of the system under test (SUT) and additionally a fault model $\delta$, which defines the coverage goal in terms of a class of faults for which the tests shall cause a specification violation. Both the specification and the coverage goal are expressed in Linear Temporal Logic (LTL) [46]. By default, our approach supports the detection of transient and permanent faults and distinguishes four fault occurrence frequencies: faults that occur at least (1) once, (2) repeatedly, (3) from some point on, or (4) permanently. Besides the four default fault occurrence frequencies, a user can also provide a custom frequency using LTL. Our approach then automatically synthesizes a test strategy to reveal a fault for the lowest frequency possible. Such a test strategy guarantees to cause a specification violation if the fault occurs with the defined fault occurrence (and all higher fault occurrence frequencies) and the test is executed long enough. Although test oracles can be synthesized from the specification $\varphi$, in this paper, we do not explicitly consider test oracle synthesis, but assume that the oracles are available or manually generated for the test strategies.

Under the hood, reactive synthesis [47] with partial information [33] is used, which provides strong guarantees about all uncertainties: if synthesis is successful and if the computed tests are executed long enough, they reveal all faults from the fault model for every realization of the specification and every behavior of the uncontrollable part of the system’s environment. Uncontrollable environment aspects can be seen as part of the system for the purpose of testing. Finally, existing techniques from runtime verification [6] can be used to build an oracle that checks the system behavior against the specification while tests are executed.^1^

This paper is an extension of [10]. In summary, this paper presents the following contributions:

- An approach to compute adaptive test strategies for reactive systems from temporal specifications that provide implementation freedom. The tests are guaranteed to reveal certain bugs for *every* realization of the specification.
- The underlying theory is considered in detail, i.e., we show that the approach is sound and complete for many interesting cases and provide additional solutions for other cases that may arise in practice.
- A proof of concept tool, called PARTYStrategy,^2^ that is capable of generating multiple different test strategies, implemented on top of the synthesis tool PARTY [31].
- A post-processing procedure to generalize a test strategy by eliminating input constraints not necessary to guarantee a coverage goal.
- A case study with a safety-critical software component of a real-world satellite system developed in the German Aerospace Center (DLR). We specify the system in LTL, synthesize test strategies, and evaluate the generated adaptive test strategies using code coverage and mutation coverage metrics. Our synthesized test strategies increase both the mutation coverage as well as the code coverage of random test cases by activating behaviors that require complex input sequences that are unlikely to be produced by random testing.

The remainder of this paper is organized as follows: Sect. 2 illustrates our approach and presents a motivating example. Section 3 discusses related work. Section 4 gives preliminaries and notation. Our test case generation approach is then worked out in detail in Sect. 5. Section 6 presents the case study and discusses results. Section 7 concludes.

## Motivating example

**Fig. 2:**
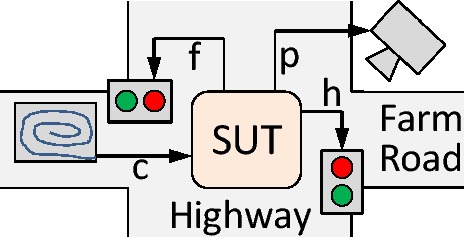
Traffic light example

Let us develop a traffic light controller for the scenario depicted in Fig. 2. For this highway and farmroad crossing, the controller’s Boolean input signal *c* describes whether a car is idling at the farmroad. Boolean outputs *h* and *f* control the highway and farmroad traffic lights respectively, where a value of $\mathsf {true}$ means a green light. Output *p* controls a camera that takes a picture if a car on the farmroad makes a fast start, i.e., races off immediately when the farmroad light turns green. The controller then should implement the following critical properties:

1. The traffic lights must never be green simultaneously.
2. If a car is waiting at the farmroad, *f* eventually turns $\mathsf {true}$.
3. If no car is waiting at the farmroad, *h* eventually becomes $\mathsf {true}$.
4. A picture is taken if a car on the farmroad makes a fast start.

We model the four properties in Linear Temporal Logic (LTL) [46] as

1 $$\begin{aligned} \varphi _1&= {{\,\mathrm{\mathsf {G}}\,}}(\lnot \mathsf {f}\vee \lnot \mathsf {h}) \end{aligned}$$

2 $$\begin{aligned} \varphi _2&={{\,\mathrm{\mathsf {G}}\,}}(\mathsf {c}\rightarrow {{\,\mathrm{\mathsf {F}}\,}}\mathsf {f}) \end{aligned}$$

3 $$\begin{aligned} \varphi _3&={{\,\mathrm{\mathsf {G}}\,}}(\lnot \mathsf {c}\rightarrow {{\,\mathrm{\mathsf {F}}\,}}\mathsf {h}) \end{aligned}$$

4 $$\begin{aligned} \varphi _4&={{\,\mathrm{\mathsf {G}}\,}}\bigl ((\lnot \mathsf {f}\wedge {{\,\mathrm{\mathsf {X}}\,}}(\mathsf {c}\wedge \mathsf {f}\wedge {{\,\mathrm{\mathsf {X}}\,}}\lnot \mathsf {c})) \leftrightarrow {{\,\mathrm{\mathsf {X}}\,}}{{\,\mathrm{\mathsf {X}}\,}}\mathsf {p}\bigr ) \end{aligned}$$

where the operator ${{\,\mathrm{\mathsf {G}}\,}}$ denotes *always*, ${{\,\mathrm{\mathsf {F}}\,}}$ denotes *eventually*, and ${{\,\mathrm{\mathsf {X}}\,}}$ denotes *in the nextstep*.

The resulting specification is then:

$$\begin{aligned} \varphi = \varphi _1 \wedge \varphi _2 \wedge \varphi _3 \wedge \varphi _4 \end{aligned}$$

To compute a test strategy (only from the specification) that enforces a specification violation by the system under the existence of a certain fault (or class of faults), we have some requirements for our approach.

*Enforcing test objectives* To mitigate scalability issues, we compute test cases directly from the specification $\varphi$. Note that $\varphi$ focuses on the desired properties only, and allows for plenty of implementation freedom. Our goal is to compute tests that *enforce* certain coverage objectives *independent* of this implementation freedom. Some uncertainties about the SUT behavior may actually be rooted in uncontrollable environment aspects (such as weather conditions) rather than implementation freedom inside the system. But for our testing approach, this makes no difference. We can force the farmroad’s traffic light to turn green ($\mathsf {f}$ = $\mathsf {true}$) by relying on a correct implementation of Property 2 and setting $\mathsf {c}$ = $\mathsf {true}$. Depending on how the system is implemented, $\mathsf {f}$ = $\mathsf {true}$ might also be achieved by setting $\mathsf {c}$ = $\mathsf {false}$ all the time, but this is not guaranteed.

*Adaptive test strategies* Certain test goals may not be enforceable with a static input sequence. For our example, for $\mathsf {p}$ to be $\mathsf {true}$, a car must do a fast start. Yet, the specification does not prescribe the exact point in time when the traffic light turns to green. We thus synthesize *adaptive* test strategies that guide the controller’s inputs based on the previous inputs and outputs and, therefore, can take advantage of situational possibilities by exploiting previous system behavior.

**Fig. 3:**
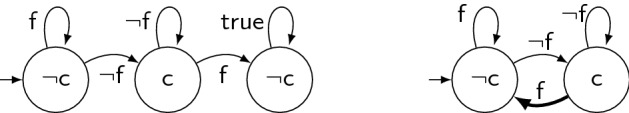
Two adaptive test strategies for the traffic light controller: on the left, $\mathcal {T}_1$ that enforces $\mathsf {p}$ = $\mathsf {true}$ once. On the right, $\mathcal {T}_2$ that enforces $\mathsf {p}$ = $\mathsf {true}$ infinitely often

Figure 3 shows a test strategy $\mathcal {T}_1$ (on the left) to reach $\mathsf {p}$ = $\mathsf {true}$, illustrated as a state machine. States are labeled by the value of controller *input* $\mathsf {c}$ (which is an *output of the test strategy*$\mathcal {T}_1$). Edges represent transitions and are labeled with conditions on observed output values (since the SUT’s outputs are inputs for the test strategy). First, $\mathsf {c}$ is set to $\mathsf {false}$ to provoke $\mathsf {h}$ = $\mathsf {true}$ via Property 3, implying $\mathsf {f}$ = $\mathsf {false}$ via Property 1. As soon as this happens, the strategy traverses to the middle state, setting $\mathsf {c}$ = $\mathsf {true}$ in order to have $\mathsf {f}$ = $\mathsf {true}$ eventually (Property 2). As soon as $\mathsf {f}$ switches from $\mathsf {false}$ to $\mathsf {true}$, $\mathcal {T}_1$ sets $\mathsf {c}$ = $\mathsf {false}$ in the rightmost state to trigger a picture (Property 4). A system with a permanent stuck-at-0 fault at signal $\mathsf {p}$ is unable to satisfy the specification and the resulting violation can be detected by a runtime verification technique.

*Coverage objectives* We follow a fault-centered approach to define the test objectives to enforce. The user defines a class of (potentially transient) faults. Our approach then computes adaptive test strategies (in form of state machines) that detect these faults. For a permanent stuck-at-0 fault at signal $\mathsf {p}$, our approach could produce the test strategy $\mathcal {T}_1$ from the previous paragraph: for any correct implementation of $\varphi$, the strategy enforces $\mathsf {p}$ becoming $\mathsf {true}$ at least once. Thus, a faulty version where $\mathsf {p}$ is always $\mathsf {false}$ necessarily violates the specification, which can be detected [6] during test strategy execution. The test strategy $\mathcal {T}_2$, as shown on the right of Fig. 3, is even more powerful since it also reveals stuck-at-0 faults for $\mathsf {p}$ that occur not always but only from some point in time onwards. The difference to $\mathcal {T}_1$ is mainly in the bold transition, which makes $\mathcal {T}_2$ enforce $\mathsf {p}$ = $\mathsf {true}$ infinitely often rather than only once. Our approach distinguishes four fault occurrence frequencies (a fault occurs at least once, infinitely often, from some point on, or always) and synthesizes test strategies for the lowest one for which this is possible.

## Background and related work

*Fault-based testing* Fault-based test case generation methods that use the concept of mutation testing [29] seed simple faults into a system implementation (or model) and compute tests that uncover these faults. Two hypotheses support the value of such tests. The Competent Programmer Hypothesis [1, 16] states that implementations are mostly close to correct. The Coupling Effect [16, 41] states that tests that detect simple faults are also sensitive to more complex faults. Our approach also relies on these hypotheses. However, in contrast to most existing work that considers permanent faults and deterministic system descriptions that define behavior unambiguously, our approach can deal with transient faults and focuses on uncovering faults in *every* implementation of a given LTL [46] specification (and all behaviors of the uncontrollable part of the system’s environment).

*Adaptive tests* If the behavior of the system or the uncontrollable part of the environment is not fully specified, tests may have to react to observed behavior at runtime to achieve their goals. Many testing theories and test case generation algorithms from specifications of labelled transition systems have been developed. Tretmans [49], for instance, proposed a testing theory analogous to the theory of testing equivalence and preorder for labelled transition systems under the assumption that an implementation communicates with its environment via inputs and outputs. Adaptive tests have been studied by Hierons [28] from a theoretical perspective, relying on fairness assumptions (every non-deterministic behavior is exhibited when trying often enough) or probabilities. Petrenko et al. compute adaptive tests for trace inclusion [43–45] or equivalence [35, 42, 44] from a specification given as non-deterministic finite state machine, also relying on fairness assumptions. Our work makes no such assumptions but considers the SUT to be fully antagonistic. Aichernig et al. [2] present a method to compute adaptive tests from (non-deterministic) UML state machines. Starting from an initial state, a trace to a goal state, the state that shall be covered by the resulting test case, is searched for every possible system behavior, issuing inconclusive verdicts only if the goal state is not reachable any more. Our approach uses reactive synthesis to enforce reaching the testing goal for all implementations if this is possible.

*Testing as a game* Yannakakis [52] points out that testing reactive systems can be seen as a game between two players: the tester providing inputs and trying to reveal faults, and the SUT providing outputs and trying to hide faults. The tester can only observe outputs and has thus partial information about the SUT. The goal is to find a strategy for the tester that wins against every SUT. The underlying complexities are studied by Alur et al. [3] in detail. Our work builds upon reactive synthesis [47] (with partial information [33]), which can also be seen as a game. However, we go far beyond the basic idea. We combine the game concept with user-defined fault models, work out the underlying theory, optimize the faults sensitivity in the temporal domain, and present a realization and experiments for LTL [46]. Nachmanson et al. [40] synthesize game strategies as tests for non-deterministic software models, but their approach is not fault-based and focuses on simple reachability goals. A variant of their approach considers the SUT to behave probabilistically with known probabilities [40]. The same model is also used in [8]. Test strategies for reachability goals are also considered by David et al. [13] for timed automata.

*Vacuity detection* Several approaches [5, 7, 34] aim at finding cases where a temporal specification is trivially satisfied (e.g., because the left side of an implication is false). Good tests avoid such vacuities to challenge the SUT. The method by Beer et al. [7] can produce witnesses that satisfy the specification non-vacuously, which can serve as tests. Our approach avoids vacuities by requiring that certain faulty SUTs violate the specification.

*Testing with a model checker* Model checkers can be utilized to compute tests from temporal specifications [25]. The method by Fraser and Ammann [22] ensures that properties are not vacuously satisfied and that faults propagate to observable property violations (using finite-trace semantics for LTL). Tan et al. [50] also define and apply a coverage metric based on vacuity for LTL. Ammann et al. [4] create tests from CTL [12] specifications using model mutations. All these methods assume that a deterministic system model is available in addition to the specification. Fraser and Wotawa [23] also consider non-deterministic models, but issue inconclusive verdicts if the system deviates from the behavior foreseen in the test case. In contrast, we search for test strategies that achieve their goal for *every* realization of the specification. Boroday et al. [11] aim for a similar guarantee (calling it *strong test cases*) using a model checker, but do not consider adaptive test cases, and use a finite state machine as a specification.

*Synthesis of test strategies* Bounded synthesis [21] aims for finding a system implementation of minimal size in the number of states. Symbolic procedures based on binary decision diagrams [18] and satisfiability solving [31] exist. In our setting, we do not synthesize an implementation of the system, but an adaptive test strategy, i.e., a controller that mimics the system’s environment to enforce a certain test goal. In contrast to a complete implementation of the controller, we strive for finding a partial implementation that assigns values only to those signals that necessarily contribute to reach the test goal. Other signals can be kept non-deterministic and either chosen during execution of the test strategy or randomized. We use a post-processing procedure that eliminates assignments from the test strategy and invokes a model-checker to verify that the test goal is still enforced. This post-processing step is conceptually similar to procedures that aim for counterexample simplification [30] and don’t care identification in test patterns [38]. Jin et al. [30] separate a counterexample trace into forced segments that unavoidably progress towards the specification violation and free segments that, if avoided, may have prevented the specification violation. Our post-processing step is similar, but instead of counterexamples, adaptive test strategies are post-processed. Miyase and Kajihara [38] present an approach to identify don’t cares in test patterns of combinational circuits. In contrast to combinational circuits, we deal with reactive systems. Instead of post-processing a complete test strategy, a partial test strategy can be directly synthesized by modifying a synthesis procedure to compute minimum satisfying assignments [17]. Although feasible, modifying a synthesis procedure requires a lot of work. Our post-processing procedure uses the synthesis procedure in a plug-and-play fashion and does not require manual changes in the synthesis procedure.

## Preliminaries and notation

*Traces* We want to test reactive systems that have a finite set $I=\{i_1,\ldots ,i_m\}$ of Boolean inputs and a finite set $O=\{o_1,\ldots ,o_n\}$ of Boolean outputs. The input alphabet is $\Sigma _I=2^I$, the output alphabet is $\Sigma _O=2^O$, and $\Sigma =2^{I\cup O}$. An infinite word ${\overline{\sigma }}$ over $\Sigma$ is an (*execution*) *trace* and the set $\Sigma ^\omega$ is the set of all infinite words over $\Sigma$.

*Linear Temporal Logic* We use *Linear Temporal Logic* (*LTL*) [46] as a specification language for reactive systems. The syntax is defined as follows: every input or output $p\in I\cup O$ is an LTL formula; and if $\varphi _1$ and $\varphi _2$ are LTL formulas, then so are $\lnot \varphi _1$, $\varphi _1 \vee \varphi _2$, ${{\,\mathrm{\mathsf {X}}\,}}\varphi _1$ and $\varphi _1 \mathbin {\mathsf {U}}\varphi _2$. We write ${\overline{\sigma }}\models \varphi$ to denote that a trace ${\overline{\sigma }}= \sigma _0 \sigma _1 \ldots \in \Sigma ^\omega$*satisfies* LTL formula $\varphi$. This is defined inductively as follows:

- $$\sigma _0 \sigma _1 \sigma _2 \ldots \models p$$ iff $$p \in \sigma _0$$,
- $${\overline{\sigma }}\models \lnot \varphi $$ iff $${\overline{\sigma }}\not \models \varphi $$,
- $${\overline{\sigma }}\models \varphi _1 \vee \varphi _2$$ iff $${\overline{\sigma }}\models \varphi _1$$ or $${\overline{\sigma }}\models \varphi _2$$,
- $$\sigma _0 \sigma _1 \sigma _2 \ldots \models {{\,\mathrm{\mathsf {X}}\,}}\varphi $$ iff $$\sigma _1 \sigma _2 \ldots \models \varphi $$, and
- $$\sigma _0 \sigma _1 \ldots \models \varphi _1 \mathbin {\mathsf {U}}\varphi _2$$ iff $$\exists j \ge 0 {{\,\mathrm{\mathbin {.}}\,}}\sigma _j \sigma _{j+1} \ldots \models \varphi _2 \wedge \forall 0 \le k < j {{\,\mathrm{\mathbin {.}}\,}}\sigma _k \sigma _{k+1} \ldots \models \varphi _1$$.

That is, ${{\,\mathrm{\mathsf {X}}\,}}\varphi$ requires $\varphi$ to hold in the *next* step, and $\varphi _1 \mathbin {\mathsf {U}}\varphi _2$ means that $\varphi _1$ must hold *until*$\varphi _2$ holds (and $\varphi _2$ must hold eventually). We also use the usual abbreviations $\varphi _1 \wedge \varphi _2 = \lnot (\lnot \varphi _1 \vee \lnot \varphi _2)$, $\varphi _1 \rightarrow \varphi _2 = \lnot \varphi _1 \vee \varphi _2$, ${{\,\mathrm{\mathsf {F}}\,}}\varphi = \mathsf {true}\mathbin {\mathsf {U}}\varphi$ (meaning that $\varphi$ must hold *eventually*), and ${{\,\mathrm{\mathsf {G}}\,}}\varphi = \lnot {{\,\mathrm{\mathsf {F}}\,}}\lnot \varphi$ ($\varphi$ must hold *always*). By $\varphi [x \leftarrow y]$ we denote the LTL formula $\varphi$ where all occurrences of *x* have been textually replaced by *y*.

*Mealy machines* We use Mealy machines to model the reactive system under test. A *Mealy machine* is a tuple $\mathcal {S}= (Q, q_0, \Sigma _I, \Sigma _O, \delta , \lambda )$, where *Q* is a finite set of states, $q_0\in Q$ is the initial state, $\delta : Q \times \Sigma _I\rightarrow Q$ is a total transition function, and $\lambda : Q \times \Sigma _I\rightarrow \Sigma _O$ is a total output function. Given the input trace ${\overline{\sigma _I}}= x_0 x_1 \ldots \in \Sigma _I^\omega$, $\mathcal {S}$ produces the output trace ${\overline{\sigma _O}}= \mathcal {S}({\overline{\sigma _I}}) = \lambda (q_0, x_0) \lambda (q_1, x_1) \ldots \in \Sigma _O^\omega$, where $q_{i+1} = \delta (q_i, x_i)$ for all $i \ge 0$. That is, in every time step *i*, the Mealy machine reads the input letter $x_i\in \Sigma _I$, responds with an output letter $\lambda (q_i, x_i) \in \Sigma _O$, and updates its state to $q_{i+1} = \delta (q_i, x_i)$. A Mealy machine can directly model synchronous hardware designs, but also other systems with inputs and outputs evolving in discrete time steps. We write ${\mathsf {Mealy}}(I,O)$ for the set of all Mealy machines with inputs $I$ and outputs $O$.

*Moore machines* We use Moore machines to describe test strategies. A *Moore machine* is a special Mealy machine with $\forall q\in Q{{\,\mathrm{\mathbin {.}}\,}}\forall x,x'\in \Sigma _I{{\,\mathrm{\mathbin {.}}\,}}\lambda (q,x) = \lambda (q,x')$. That is, $\lambda (q,x)$ is insensitive to *x*, i.e., becomes a function $\lambda : Q \rightarrow \Sigma _O$. This means that the input $x_i$ at step *i* can affect the next state $q_{i+1}$ and thus the next output $\lambda (q_{i+1})$ but not the current output $\lambda (q_i)$. We write ${\mathsf {Moore}}(I,O)$ for the set of all Moore machines with inputs $I$ and outputs $O$.

*Composition* Given Mealy machines $$\mathcal {S}_1 = (Q_1, q_{0,1}, 2^I, 2^{O_1}, \delta _1, \lambda _1) \in {\mathsf {Mealy}}(I,O_1)$$ and $$\mathcal {S}_2 = (Q_2, q_{0,2}, 2^{I\cup O_1}, 2^{O_2}, \delta _2, \lambda _2) \in {\mathsf {Mealy}}(I\cup O_1, O_2)$$, we write $$\mathcal {S}= \mathcal {S}_1 \circ \mathcal {S}_2$$ for their sequential composition $$\mathcal {S}= (Q_1 \times Q_2, (q_{0,1}, q_{0,2}), 2^I, 2^{O_1 \cup O_2},$$$$ \delta , \lambda )$$, where $$\mathcal {S}\in {\mathsf {Mealy}}(I,O_1\cup O_2)$$ with $$\delta \bigl ((q_1, q_2), x\bigr ) = \bigl (\delta _1(q_1,x), \delta _2(q_2, x \cup \lambda _1(q_1,x))\bigr )$$ and $$\lambda \bigl ((q_1, q_2), x\bigr ) = \lambda _1(q_1,x) \cup \lambda _2\bigl (q_2,x \cup \lambda _1(q_1,x)\bigr )$$. Note that $$x \in 2^I$$.

*Systems and test strategies* A *reactive system*$\mathcal {S}$ is a Mealy machine. An (*adaptive*) *test strategy* is a Moore machine $\mathcal {T}= (T, t_0, \Sigma _O, \Sigma _I, \Delta , \Lambda )$ with input and output alphabet swapped. That is, $\mathcal {T}$ produces values for input signals and reacts to values of output signals. A test strategy $\mathcal {T}$ can be *run* on a system $\mathcal {S}$ as follows. In every time step *i* (starting with $i=0$), $\mathcal {T}$ first computes the next input $x_i=\Lambda (t_i)$. Then, the system computes the output $y_i = \lambda (q_i, x_i)$. Finally, both machines compute their next state $t_{i+1} = \Delta (t_i, y_i)$ and $q_{i+1} = \delta (q_i, x_i)$. We write ${\overline{\sigma }}(\mathcal {T},\mathcal {S}) = (x_0 \cup y_0) (x_1 \cup y_1) \ldots \in \Sigma ^\omega$ for the resulting execution trace. If $\mathcal {T}= (T, t_0, 2^{O'}, \Sigma _I, \Delta , \Lambda ) \in {\mathsf {Moore}}(O', I)$ can observe only a subset $O'\subseteq O$ of the outputs, we define ${\overline{\sigma }}(\mathcal {T},\mathcal {S})$ with $t_{i+1} = \Delta (t_i, y_i \cap O')$. A *test suite* is a set $\text {TS}\subseteq {\mathsf {Moore}}(O,I)$ of adaptive test strategies.

*Realizability* A Mealy machine $\mathcal {S}\in {\mathsf {Mealy}}(I,O)$*realizes* an LTL formula $\varphi$, written , if $\forall \mathcal {M}\in {\mathsf {Moore}}(O, I) {{\,\mathrm{\mathbin {.}}\,}}{\overline{\sigma }}(\mathcal {M},\mathcal {S}) \models \varphi$. An LTL formula $\varphi$ is *Mealy-realizable* if there exists a Mealy machine that realizes it. A Moore machine $\mathcal {M}\in {\mathsf {Moore}}(I, O)$ realizes $\varphi$, written , if $\forall \mathcal {S}\in {\mathsf {Mealy}}(O, I) {{\,\mathrm{\mathbin {.}}\,}}{\overline{\sigma }}(\mathcal {M},\mathcal {S}) \models \varphi$. A *model checking procedure* checks if a given Mealy (Moore) machine $\mathcal {S}$ ($\mathcal {M}$) realizes an LTL specification $\varphi$ and returns $\mathsf {true}$ iff  () holds. We denote the call of a model checking procedure by ${\mathsf {modelcheck}}\bigl (\mathcal {S},\varphi \bigr )$ (${\mathsf {modelcheck}}\bigl (\mathcal {M},\varphi \bigr )$).

*Reactive synthesis* We use reactive synthesis [47] to compute test strategies. A *reactive (Moore, LTL) synthesis procedure* takes as input a set $I$ of Boolean inputs, a set $O$ of Boolean outputs, and an LTL specification $\varphi$ over these signals. It produces a Moore machine $\mathcal {M}\in {\mathsf {Moore}}(I, O)$ that realizes $\varphi$, or the message unrealizable if no such Moore machine exists. We denote this computation by $\mathcal {M}= {\mathsf {synt}}(I, O, \varphi )$.

*Synthesis with partial information* [33] is defined similarly, but this problem takes a subset $I' \subseteq I$ of the inputs as an additional input. As output, the synthesis procedure produces a Moore machine $\mathcal {M}' = {\mathsf {synt}}_p(I, O, \varphi , I')$ with $\mathcal {M}' \in {\mathsf {Moore}}(I', O)$ that realizes $\varphi$ while only observing the inputs $I'$, or the message unrealizable if no such Moore machine exists. We assume that both synthesis procedure, ${\mathsf {synt}}$ and ${\mathsf {synt}}_p$, can be called *incrementally* with an additional parameter $\Theta$, where $\Theta$ denotes a set of Moore machines. The incremental synthesis procedures $\mathcal {M}= {\mathsf {synt}}(I, O, \varphi , \Theta )$ and $\mathcal {M}' = {\mathsf {synt}}_p(I, O, \varphi , I', \Theta )$ compute Moore machines $\mathcal {M}$ and $\mathcal {M}^\prime$, respectively, as before but with the additional constraints that $\mathcal {M}, \mathcal {M}^\prime \not \in \Theta$.

In synthesis, we often use *assumptionsA* and *guaranteesG*. The assumptions are meant to state the requirements on the environment under which the guarantees should be met by the synthesized system. Technically, we synthesize a system $\mathcal {M}$ that fulfills the specification $A \rightarrow G$. Obviously, whenever the environment violates the assumptions, the implication is trivially satisfied and the behavior of the system is irrelevant.

For the purposes of this paper, we take synthesis as a black box. We will not describe the technical details of synthesis here but rather refer the interested reader to [9] for details.

*Fault versus failure* A Mealy machine $\mathcal {S}\in {\mathsf {Mealy}}(I,O)$ is *faulty* with respect to LTL formula $\varphi$ (specification) iff , i.e., $\exists \mathcal {M}\in {\mathsf {Moore}}(O, I) {{\,\mathrm{\mathbin {.}}\,}}{\overline{\sigma }}(\mathcal {M},\mathcal {S}) \not \models \varphi$. We call a trace ${\overline{\sigma }}(\mathcal {M},\mathcal {S})$ that uncovers a faulty behavior of $\mathcal {S}$ a *failure* and a deviation between $\mathcal {S}$ and any correct realization $\mathcal {S}^\prime$, i.e., , a *fault*. For a fixed faulty $\mathcal {S}$, there are multiple correct $\mathcal {S}^\prime$ that realize $\varphi$ and thus a fault in $\mathcal {S}$ can be characterized by multiple, different ways. As a simplification, we assume that in practice every faulty $\mathcal {S}$ is close to a correct $\mathcal {S}^\prime$ and only deviates in a simple fault. In the next section, we will show how this idea can be leveraged to determine test suites independent of the implementation and the concrete fault manifestation.

## Synthesis of adaptive test strategies

This section presents our black-box testing approach for synthesizing adaptive test strategies for reactive systems specified in LTL. First, we elaborate on the coverage objective we aim to achieve. Then we present our strategy synthesis algorithm. Finally, we discuss extensions and variants of the algorithm.

### Coverage objective for test strategy computation

**Fig. 4:**
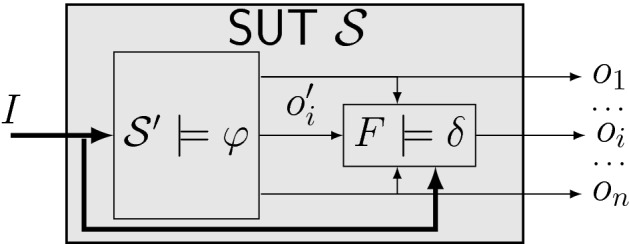
Coverage goal illustration for fault

Many coverage metrics [37] exist to assess the quality of a test suite. Since the goal in testing is to detect bugs, we follow a fault-centered approach: a test suite has high quality if it reveals certain kinds of faults in a system. In contrast to existing approaches such as mutation testing which model potential faults in a concrete implementation, we provide a novel fault model that models faults on the specification-level, agnostic of the concrete implementation. We assume that the SUT is “almost correct” and contains only simple faults that propagate to at most one output.^3^ As illustrated in Fig. 4, we formalize this assumption on specification-level and model the SUT as composed of a correct implementation $\mathcal {S}'$ of the specification $\varphi$ and a fault *F* that affects one output. In order to make our approach flexible, we allow the user to define the considered faults as an LTL formula $\delta$. Through $\delta$, the user can define both permanent and transient faults of various types. For instance, $\delta = {{\,\mathrm{\mathsf {F}}\,}}(o_i \leftrightarrow \lnot o_i')$ describes a bit-flip that occurs at least once, ${{\,\mathrm{\mathsf {G}}\,}}\!{{\,\mathrm{\mathsf {F}}\,}}\lnot o_i$ models a stuck-at-0 fault that occurs infinitely often, and ${{\,\mathrm{\mathsf {G}}\,}}({{\,\mathrm{\mathsf {X}}\,}}(o_i) \leftrightarrow o_i')$ models a permanent shift by one time step. We strive for a test suite that reveals *every* fault that satisfies $\delta$ for *every* realization of $\varphi$. This renders the test suite independent of the implementation and the concrete fault manifestation. The following definition formalizes this intuition into a coverage objective.

#### Definition 1

A test suite $\text {TS}\subseteq {\mathsf {Moore}}(O,I)$ for a system with inputs $I$, outputs $O$, and specification $\varphi$ is *universally complete*^4^ with respect to a given fault model $\delta$ iff5

That is, for every output $o_i$, system , and fault , $\text {TS}$ must contain a test strategy $\mathcal {T}$ that reveals the fault by causing a specification violation (Fig. 4). Note that the test strategies $\mathcal {T}\in \text {TS}\subseteq {\mathsf {Moore}}(O,I)$ cannot observe the signal $o_i'$. The reason is that this signal $o_i'$ does not exist in the real system implementation(s) on which we run our tests—it was only introduced to define our coverage objective.

There can be an unbounded number of system realizations  and faults . Computing a separate test strategy for each combination is thus not a viable option. We rather strive for computing only one test strategy per output variable.

#### Theorem 1

A universally complete test suite $\text {TS}\subseteq {\textsf {Moore}}(O,I)$ with respect to fault model $\delta$ exists for a system with inputs $I$, outputs $O$, and specification $\varphi$ if

6 $$\begin{aligned} \forall o_i\in O{{\,\mathrm{\mathbin {.}}\,}}\exists \mathcal {T}\in & {} {\textsf {Moore}}(O,I) {{\,\mathrm{\mathbin {.}}\,}}\forall \mathcal {S}\in {\textsf {Mealy}}(I,O\cup \{o_i'\}) {{\,\mathrm{\mathbin {.}}\,}}\nonumber \\&\qquad \qquad \qquad \qquad \qquad \qquad \qquad {\overline{\sigma }}(\mathcal {T}, \mathcal {S}) \models \bigl ((\varphi [o_i\leftarrow o_i'] \wedge \delta ) \rightarrow \lnot \varphi \bigr ). \end{aligned}$$

#### Proof

Equation 6 implies7because (a) going from $\exists \mathcal {T}\forall \mathcal {S}$ to $\forall \mathcal {S}\exists \mathcal {T}$ can only make the formula weaker, and (b)  implies ${\overline{\sigma }}(\mathcal {T}, \mathcal {S}) \models \varphi [o_i\leftarrow o_i'] \wedge \delta$ for all $\mathcal {T}$, which can only make the left side of the implication stronger. In turn, Eq. 7 is equivalent to8because for a given  and  from Eq. 8 we can define an equivalent system $\mathcal {S}=(\mathcal {S}' \circ F) \in {\mathsf {Mealy}}(I,O\cup \{o_i'\})$ for Eq. 7 such that  is satisfied. Also, for a given  from Eq. 7 we can define a corresponding  and  by stripping off different outputs.

Theorem 1 states that Eq. 6 is a sufficient condition for a universally complete test suite to exist. If it were also a necessary condition, then computing one test strategy per output signal would be enough. Unfortunately, this is not the case in general.

**Fig. 5:**
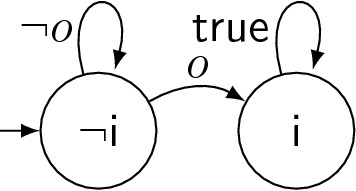
Test strategy $$\mathcal {T}_5$$

#### Example 1

Consider a system with input $I=\{i\}$, output $O=\{o\}$, and specification $\varphi = \bigl ( {{\,\mathrm{\mathsf {G}}\,}}(i \rightarrow {{\,\mathrm{\mathsf {G}}\,}}i) \wedge {{\,\mathrm{\mathsf {F}}\,}}i \bigr ) \rightarrow \bigl ( {{\,\mathrm{\mathsf {G}}\,}}(o \rightarrow {{\,\mathrm{\mathsf {G}}\,}}o) \wedge {{\,\mathrm{\mathsf {F}}\,}}o \wedge {{\,\mathrm{\mathsf {G}}\,}}(i \vee \lnot o) \bigr )$. The left side of the implication assumes that the input *i* is set to $\mathsf {true}$ at some point, after which *i* remains $\mathsf {true}$. The right side requires the same for the output *o*. In addition, *o* must not be raised while *i* is still $\mathsf {false}$. This specification is realizable (e.g., by always setting $o=i$). The test suite $\text {TS}= \{\mathcal {T}_5\}$ with $\mathcal {T}_5$ shown in Fig. 5 is universally complete with respect to fault model $\delta = {{\,\mathrm{\mathsf {F}}\,}}(o \leftrightarrow \lnot o')$, which requires the output to flip at least once: as long as *i* is $\mathsf {false}$, any correct system implementation  must keep the output $o'=\mathsf {false}$. Eventually,  must flip the output *o* to $\mathsf {true}$. When this happens, *i* is set to $\mathsf {true}$ by $\mathcal {T}_5$ so that the resulting trace ${\overline{\sigma }}(\mathcal {T}, \mathcal {S}' \circ F)$ violates $\varphi$. Still, Eq. 6 is $\mathsf {false}$.^5^ Strategy $\mathcal {T}_5$ does not satisfy Eq. 6 because for the system $\mathcal {S}\in {\mathsf {Mealy}}(\{i\},\{o,o'\})$ that sets $o'=\mathsf {true}$ and $o=\mathsf {false}$ in all time steps, we have ${\overline{\sigma }}(\mathcal {T}_5, \mathcal {S}) \models \bigl (\varphi [o_i\leftarrow o_i'] \wedge \delta \wedge \varphi \bigr )$. The reason is that *i* stays $\mathsf {false}$, so $\varphi [o_i\leftarrow o_i']$ and $\varphi$ are vacuously satisfied by ${\overline{\sigma }}(\mathcal {T}_5, \mathcal {S})$. The formula $\delta$ is satisfied because $o \leftrightarrow \lnot o'$ holds in all time steps. Thus, $\mathcal {S}$ is a counterexample to $\mathcal {T}_5$ satisfying Eq. 6. Similar counterstrategies exist for all other test strategies.

The fact that Eq. 6 is not a necessary condition for a universally complete test suite to exist is somewhat surprising, especially in the light of the following two lemmas. Based on these lemmas, the subsequent propositions will show that Eq. 6 is both sufficient and necessary (i.e., one test per output is enough) for many interesting cases.

The following lemma, which is based on the determinacy of complete-information games, states that the following two conditions are equivalent: (1) there is a single test strategy that shows a fault in any implementation and (2) for any implementation there is a strategy that shows the fault. This means that in certain settings, a single test strategy suffices to find a fault.

#### Lemma 1

For every LTL specification $\psi$ over some inputs $I$ and outputs $O$, we have that $\exists \mathcal {T}\in {\textsf {Moore}}(O, I) {{\,\mathrm{\mathbin {.}}\,}}\forall \mathcal {S}\in {\textsf {Mealy}}(I, O) {{\,\mathrm{\mathbin {.}}\,}}{\overline{\sigma }}(\mathcal {T}, \mathcal {S}) \models \psi$ holds if and only if $\forall \mathcal {S}\in {\textsf {Mealy}}(I, O) {{\,\mathrm{\mathbin {.}}\,}}\exists \mathcal {T}\in {\textsf {Moore}}(O, I) {{\,\mathrm{\mathbin {.}}\,}}{\overline{\sigma }}(\mathcal {T}, \mathcal {S}) \models \psi$ holds.

#### Proof

Synthesis from LTL specifications under complete information is (finite memory) determined [36], which means that either $$\exists \mathcal {T}\in {\mathsf {Moore}}(O, I) {{\,\mathrm{\mathbin {.}}\,}}$$$$\forall \mathcal {S}\in {\mathsf {Mealy}}(I, O) {{\,\mathrm{\mathbin {.}}\,}}{\overline{\sigma }}(\mathcal {T}, \mathcal {S}) \models \psi $$ or $$\exists \mathcal {S}\in {\mathsf {Mealy}}(I, O) {{\,\mathrm{\mathbin {.}}\,}}\forall \mathcal {T}\in {\mathsf {Moore}}(O, I) {{\,\mathrm{\mathbin {.}}\,}}{\overline{\sigma }}(\mathcal {T}, \mathcal {S}) \models \lnot \psi $$ holds, but not both. Less formally we can say that either there exists a test strategy $$\mathcal {T}$$ that satisfies $$\psi $$ for all systems $$\mathcal {S}$$, or there exists a system $$\mathcal {S}$$ that can violate $$\psi $$ for all test strategies $$\mathcal {T}$$. From that, it follows that

The second lemma is again limited to perfect information. It states that the following two conditions are equivalent: (1) for any system that fulfills an assumption *A*, there is a test strategy that elicits behavior satisfying a guarantee *G* and (2) for any system there is a test strategy that elicits behavior satisfying the LTL property $A \rightarrow G$. This lemma implies that in the case of complete information, an LTL synthesis tool suffices.

#### Lemma 2

For all LTL specifications *A*, *G* over inputs $I$ and outputs $O$, we have that910

#### Proof

Direction $\Rightarrow$: We show that Eq. 10 being $\mathsf {false}$ contradicts with Eq. 9 being $\mathsf {true}$.Direction $\Leftarrow$: Using the LTL semantics, we can rewrite ${\overline{\sigma }}(\mathcal {T}, \mathcal {S}) \models A \rightarrow G$ in Eq. 10 as $\bigl ({\overline{\sigma }}(\mathcal {T}, \mathcal {S}) \models A\bigr ) \rightarrow \bigl ({\overline{\sigma }}(\mathcal {T}, \mathcal {S}) \models G\bigr )$. Since  implies ${\overline{\sigma }}(\mathcal {T}', \mathcal {S}) \models A$ for every $\mathcal {T}' \in {\mathsf {Moore}}(I, O)$, the assumption in Eq. 9 is not weaker, so Eq. 9 is not stronger.

Yet, in our setting, test strategies $\mathcal {T}\in {\mathsf {Moore}}(O,I)$ have incomplete information about the system $\mathcal {S}\in {\mathsf {Mealy}}(I,O\cup \{o_i'\})$ because they cannot observe $o_i'$. Still, $\mathcal {T}$ must enforce $(\varphi [o_i\leftarrow o_i'] \wedge \delta ) \rightarrow \lnot \varphi ,$ which refers to this hidden signal. Thus, Lemma 1 and 2 cannot be applied to Eq. 6 in general. However, in cases where there is (effectively) no hidden information, the lemmas can be used to prove that Eq. 6 is both a necessary and a sufficient condition for a universally complete test suite to exist. The following propositions show that this holds for many cases of practical interest.

The intuitive reason is that $\varphi [o_i\leftarrow o_i']$ can be rewritten to $\varphi [o_i\leftarrow \psi ]$ in Eq. 6, which eliminates the hidden signal such that Lemmas 1 and 2 can be applied.

#### Proposition 1

Given a fault model of the form $\delta = {{\,\mathrm{\mathsf {G}}\,}}(o_i' \leftrightarrow \psi )$, where $\psi$ is an LTL formula over $I$ and $O$, a universally complete test suite $\text {TS}\subseteq {\textsf {Moore}}(O,I)$ with respect to $\delta ,I,O$, and $\varphi$ exists if and only if Eq. 6 holds.

#### Proof

$\varphi [o_i\leftarrow o_i'] \wedge {{\,\mathrm{\mathsf {G}}\,}}(o_i' \leftrightarrow \psi )$ is equivalent to $\varphi [o_i\leftarrow \psi ] \wedge {{\,\mathrm{\mathsf {G}}\,}}(o_i' \leftrightarrow \psi )$. Thus, Eq. 6 becomes

$$\begin{aligned} \forall o_i\in O{{\,\mathrm{\mathbin {.}}\,}}\exists \mathcal {T}\in {\mathsf {Moore}}(O,I) {{\,\mathrm{\mathbin {.}}\,}}\forall \mathcal {S}\in & {} {\mathsf {Mealy}}(I,O\cup \{o_i'\}) {{\,\mathrm{\mathbin {.}}\,}}\\&{\overline{\sigma }}(\mathcal {T}, \mathcal {S}) \models (\varphi [o_i\leftarrow \psi ] \wedge {{\,\mathrm{\mathsf {G}}\,}}(o_i' \leftrightarrow \psi )) \rightarrow \lnot \varphi , \end{aligned}$$

which is equivalent to

$$\begin{aligned} \forall o_i\in O{{\,\mathrm{\mathbin {.}}\,}}\exists \mathcal {T}\in {\mathsf {Moore}}(O,I) {{\,\mathrm{\mathbin {.}}\,}}\forall \mathcal {S}\in & {} {\mathsf {Mealy}}(I,O) {{\,\mathrm{\mathbin {.}}\,}}\\&{\overline{\sigma }}(\mathcal {T}, \mathcal {S}) \models \varphi [o_i\leftarrow \psi ] \rightarrow \lnot \varphi . \end{aligned}$$

Because of the ${{\,\mathrm{\mathsf {G}}\,}}$ operator, a unique value for $o_i'$ exist in all time steps and thus, $o_i'$ is just an abbreviation for $\psi$. Whether this abbreviation $o_i'$ is available as output of $\mathcal {S}$ or not is irrelevant, because $\mathcal {T}$ cannot observe $o_i'$ anyway. Since $o_i'$ no longer occurs, Lemmas 1 and 2 can be applied to prove equivalence between Eq. 6 andAs $\mathcal {T}$ cannot observe $o_i'$, it is irrelevant whether the truth value of $\psi$ is available as additional output $o_i'$ of $\mathcal {S}$ or not. Hence, the above formula is equivalent toandi.e., to Eq. 7. The remaining steps can be taken from the proof of Theorem 1.

Proposition 1 entails that computing one test strategy per output $o_i\in O$ is enough for fault models such as permanent bit flips (defined by $\delta = {{\,\mathrm{\mathsf {G}}\,}}(o_i' \leftrightarrow \lnot o_i)$).

#### Proposition 2

If the fault model $\delta$ does not reference $o_i'$, a universally complete test suite $\text {TS}\subseteq {\textsf {Moore}}(O,I)$ with respect to $\delta ,I,O$, and $\varphi$ exists iff Eq. 6 holds.

#### Proof

We show that Eq. 6 holds if and only if Eq. 7 holds. The remaining steps have already been proven for Theorem 1.

#### Lemma 3

Equation 6 holds if and only if

11 $$\begin{aligned} \begin{aligned} \forall o_i\in O{{\,\mathrm{\mathbin {.}}\,}}\exists \mathcal {T}\in {\textsf {Moore}}(O,I) {{\,\mathrm{\mathbin {.}}\,}}\forall \mathcal {S}\in {\textsf {Mealy}}(I,O) {{\,\mathrm{\mathbin {.}}\,}}\\ {\overline{\sigma }}(\mathcal {T}, \mathcal {S}) \models \delta \rightarrow \lnot \varphi . \end{aligned} \end{aligned}$$

#### Proof

Direction $\Leftarrow$ is obvious because Eq. 6 contains stronger assumptions (and $\forall \mathcal {S}\in {\mathsf {Mealy}}(I,O)$ can be changed to $\forall \mathcal {S}\in {\mathsf {Mealy}}(I,O\cup \{o_i'\})$ in Eq. 11 because $\delta \rightarrow \lnot \varphi$ does not contain $o_i'$).

Direction $\Rightarrow$: We show that Eq. 11 being $\mathsf {false}$ contradicts with Eq. 6 being $\mathsf {true}$.1213141516171819which contradicts Eq. 6. (13)$\Leftrightarrow$(14) holds because of Lemma 1 and (Eq. 15)$\Leftrightarrow$ (Eq. 16) holds because $\delta \wedge \varphi$ does not contain $o_i'$, so $\mathcal {S}'$ can be $\mathcal {S}$ with $o_i' \leftrightarrow o_i$. (Eq. 17)$\Leftrightarrow$ (Eq. 18) holds because of Lemma 1. Finally, (Eq. 18) implies (Eq. 19) because $\mathcal {T}$ has less information in (Eq. 19).

#### Lemma 4

Equation 11 holds if and only if Eq. 7 holds.

#### Proof

Direction $\Rightarrow$: is obvious because Eq. 11 is equivalent to Eq. 6 (Lemma 3) and Eq. 6 implies Eq. 7 (see proof for Theorem 1).

Direction $\Leftarrow$: we show that Eq. 11 being $\mathsf {false}$ contradicts Eq. 7 being $\mathsf {true}$. Equation 11 being $\mathsf {false}$ implies Eq. 16 (see above). As  implies  for all $\mathcal {T}\in {\mathsf {Moore}}(O\cup \{o_i'\},I)$ and thus also for all $\mathcal {T}\in {\mathsf {Moore}}(O,I)$, Eq. 7 cannot hold.

Thus, the assumption  can be dropped from Eq. 5 if the fault model does not reference $o_i'$. Correspondingly, ${\overline{\sigma }}(\mathcal {T}, \mathcal {S}) \models \bigl ((\varphi [o_i\leftarrow o_i'] \wedge \delta ) \rightarrow \lnot \varphi \bigr )$ simplifies to ${\overline{\sigma }}(\mathcal {T}, \mathcal {S}) \models (\delta \rightarrow \lnot \varphi )$ in Eq. 6. Since $o_i'$ is now gone, Lemmas 1 and 2 apply. In general, the assumption  is needed to prevent a faulty system  from compensating the fault  such that . E.g., for $I=\emptyset$, $O=\{o\}$, $\varphi ={{\,\mathrm{\mathsf {G}}\,}}o$ and $\delta = {{\,\mathrm{\mathsf {G}}\,}}(o \leftrightarrow \lnot o')$, Eq. 5 would be $\mathsf {false}$ without  because there exists an $\mathcal {S}'$ that always sets $o'=\mathsf {false}$, in which case $\mathcal {S}' \circ F$ has *o* correctly set to $\mathsf {true}$. However, if $\delta$ does not reference $o'$, such a fault compensation is not possible.

Proposition 2 applies to permanent or transient stuck-at-0 or stuck-at-1 faults (e.g., $\delta ={{\,\mathrm{\mathsf {F}}\,}}\lnot o_i$ or $\delta ={{\,\mathrm{\mathsf {G}}\,}}\!{{\,\mathrm{\mathsf {F}}\,}}o_i$), but also to faults where $o_i$ keeps its previous value (e.g., $\delta ={{\,\mathrm{\mathsf {F}}\,}}(o_i\leftrightarrow {{\,\mathrm{\mathsf {X}}\,}}o_i$) or takes the value of a different input or output (e.g., $\delta ={{\,\mathrm{\mathsf {G}}\,}}\!{{\,\mathrm{\mathsf {F}}\,}}(o_i \leftarrow i_3)$). Together with Proposition 1, it shows that computing one test strategy per output is enough for many interesting fault models. Finally, even if neither Propositions 1 nor 2 applies, computing one test strategy per output may still suffice for the concrete $\varphi$ and $\delta$ at hand. In the next section, we thus rely on Eq. 6 to compute one test strategy per output in order to obtain universally complete test suites.

### Test strategy computation

*Basic idea* Our test case generation approach builds upon Theorem 1: for every output $o_i\in O$, we want to find a test strategy $\mathcal {T}_i \in {\mathsf {Moore}}(O,I)$ such that $\forall \mathcal {S}\in {\mathsf {Mealy}}(I,O\cup \{o_i'\}) {{\,\mathrm{\mathbin {.}}\,}}{\overline{\sigma }}(\mathcal {T}_i, \mathcal {S}) \models (\varphi [o_i\leftarrow o_i'] \wedge \delta ) \rightarrow \lnot \varphi$ holds. Recall from Sect. 4 that a synthesis procedure $\mathcal {M}= {\mathsf {synt}}_p(I, O, \psi , I',\Theta )$ with partial information computes a Moore machine $\mathcal {M}\in {\mathsf {Moore}}(I', O) {\setminus } \Theta$ with $I'\subseteq I$ such that a certain LTL objective $\psi$ is enforced in all environments, i.e., $\forall \mathcal {S}\in {\mathsf {Mealy}}(O, I) {{\,\mathrm{\mathbin {.}}\,}}{\overline{\sigma }}(\mathcal {M},\mathcal {S}) \models \psi$. If no such $\mathcal {M}$ exists, ${\mathsf {synt}}_p$ returns unrealizable . Also recall that a test strategy is a Moore machine with input and output signals swapped. We can thus call $\mathcal {T}_i := {\mathsf {synt}}_p\bigl (O\cup \{o_i'\}, I, (\varphi [o_i\leftarrow o_i'] \wedge \delta ) \rightarrow \lnot \varphi , O,\Theta \bigr )$ for every output $o_i \in O$ in order to obtain a universally complete test suite with respect to fault model $\delta$ for a system with inputs $I$, outputs $O$, and specification $\varphi$. If ${\mathsf {synt}}_p$ succeeds (does not return unrealizable ) for all $o_i \in O$, the resulting test suite $\text {TS}= \{\mathcal {T}_i \mid o_i\in O\}$ is guaranteed to be universally complete. However, since Theorem 1 only gives a sufficient but not a necessary condition, this procedure may fail to find a universally complete test suite, even if one exists, in general. In cases where Propositions 1 or 2 applies, it is both sound and complete, though.

**Fig. 6:**
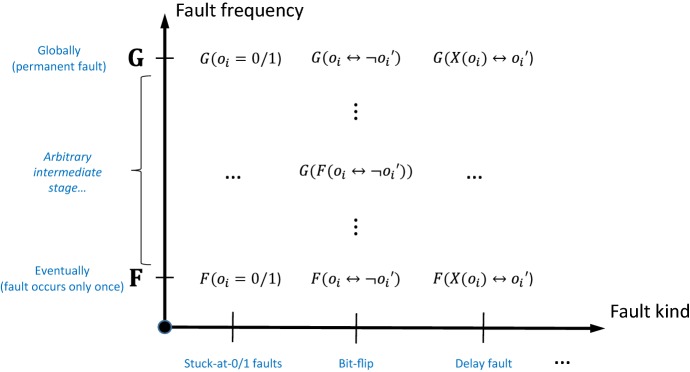
Examples of different coverage objectives

*Fault models* In order to simplify the user input, we split the fault model $\delta$ in our coverage objective from Definition 1 into two parts: the fault kind $\kappa$ and the fault frequency $\mathsf {frq}$ (Fig. 6). The fault kind $\kappa$ is an LTL formula that is given by the user and defines *which* faults we consider. For instance, $\kappa =\lnot o_i$ describes a stuck-at-0 fault, $\kappa =o_i \leftrightarrow \lnot o_i'$ defines a bit-flip, and $\kappa =o_i' \leftrightarrow {{\,\mathrm{\mathsf {X}}\,}}o_i$ describes a delay by one time step. The fault frequency $\mathsf {frq}$ describes *how often* a fault of the specified kind occurs, and is chosen by our algorithm, unless it is specified by the user. We distinguish 4 fault frequencies, which we describe using temporal LTL operators.

- Fault frequency ${{\,\mathrm{\mathsf {G}}\,}}$ means that the fault is permanent.
- Frequency ${{\,\mathrm{\mathsf {F}}\,}}\! {{\,\mathrm{\mathsf {G}}\,}}$ means that the fault occurs from some time step *i* on permanently. Yet, we do not make any assumptions about the precise value of *i*.
- Frequency ${{\,\mathrm{\mathsf {G}}\,}}\! {{\,\mathrm{\mathsf {F}}\,}}$ states that the fault strikes infinitely often, but not when exactly.
- Frequency ${{\,\mathrm{\mathsf {F}}\,}}$ means that the fault occurs at least once.

The fault model $\delta$ is then defined as $\delta =\mathsf {frq}(\kappa )$. Note that there is a natural order among our 4 fault frequencies: a fault of kind $\kappa$ that occurs permanently (frequency ${{\,\mathrm{\mathsf {G}}\,}}$) is just a special case of the same fault $\kappa$ occurring from some point onwards (frequency ${{\,\mathrm{\mathsf {F}}\,}}\! {{\,\mathrm{\mathsf {G}}\,}}$), which is in turn a special case of $\kappa$ occurring infinitely often (frequency ${{\,\mathrm{\mathsf {G}}\,}}\! {{\,\mathrm{\mathsf {F}}\,}}$), which is a special case of $\kappa$ occurring at least once. Thus, a test strategy that reveals a fault that occurs at least once (without knowing when) will also reveal a fault that occurs infinitely often, etc. We say that ${{\,\mathrm{\mathsf {F}}\,}}$ is the lowest and ${{\,\mathrm{\mathsf {G}}\,}}$ is the highest fault frequency. In our approach, we thus compute test strategies to detect faults at the lowest frequency for which a test strategy can be found. Figure 6 presents different examples of the fault model.
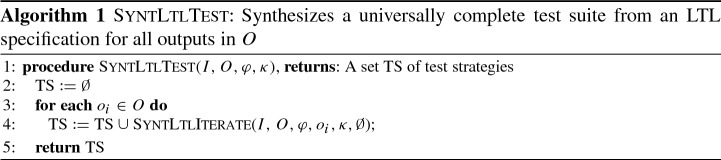

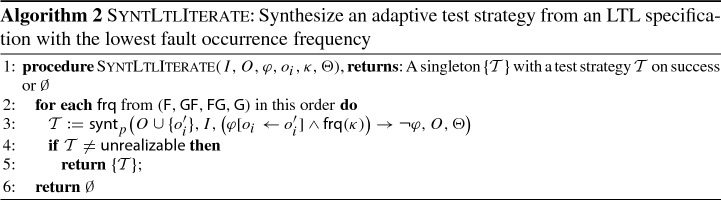
*Algorithm* The procedure SyntLtlTest in Algorithm 1 formalizes our approach using the procedure SyntLtlIterate in Algorithm 2 as a helper. The input consists of (1) the inputs $I$ of the SUT, (2) the outputs $O$ of the SUT, (3) an LTL specification $\varphi$ of the SUT, and (4) a fault kind $\kappa$. The result of SyntLtlTest is a test suite $\text {TS}$. The algorithm iterates over all outputs $o_i \in O$ (Line 3) and invokes the procedure SyntLTLIterate (Line 4). The procedure SyntLTLIterate then iterates over the 4 fault frequencies (Line 2), starting with the lowest one, and attempts to compute a strategy to reveal a fault (Line 3). If such a strategy exists, it is returned to Algorithm 1 and added to $\text {TS}$. Otherwise, the procedures proceeds with the next higher fault frequency.

*Sanity checks* Note that our coverage goal in Eq. 5 is vacuously satisfied by any test suite if $\varphi$ or $\delta$ is unrealizable. The reason is that the test suite must reveal *every* fault *F* realizing $\delta$ for *every* system $\mathcal {S}'$ realizing $\varphi$. If there is no such fault or system, this is trivial. As a sanity check, we thus test the (Mealy) realizability of $\varphi$ and ${{\,\mathrm{\mathsf {G}}\,}}\kappa$ before starting Algorithm 1 (because if ${{\,\mathrm{\mathsf {G}}\,}}\kappa$ is realizable, then so are ${{\,\mathrm{\mathsf {F}}\,}}\!{{\,\mathrm{\mathsf {G}}\,}}\kappa$, ${{\,\mathrm{\mathsf {G}}\,}}\!{{\,\mathrm{\mathsf {F}}\,}}\kappa$ and ${{\,\mathrm{\mathsf {F}}\,}}\kappa$).

*Handling unrealizability* If, for some output, Line 3 of Algorithm 2 returns unrealizable for the highest fault frequency $\mathsf {frq}={{\,\mathrm{\mathsf {G}}\,}}$, we print a warning and suggest that the user examines these cases manually. There are two possible reasons for unrealizability. First, due to limited observability, we do not find a test strategy although one exists (see Example 1). Second, no test strategy exists because there is some  and  such that the composition $\mathcal {S}= \mathcal {S}' \circ F$ (see Fig. 4) is correct, i.e., . In other words, for some realization, adding the fault may result in an equivalent mutant in the sense that the specification is still satisfied. For example, in case of a stuck-at-0 fault model, there may exist a realization of the specification that has the considered output $o_i\in O$ fixed to $\mathsf {false}$. Such a high degree of underspecification is at least suspicious and may indicate unintended vacuities [7] in the specification $\varphi$, which should be investigated manually. If Proposition 1 or 2 applies, or if ${\mathsf {synt}}\bigl (O\cup \{o_i'\}, I, \bigl (\varphi [o_i\leftarrow o_i'] \wedge {{\,\mathrm{\mathsf {G}}\,}}(\kappa )\bigr ) \rightarrow \lnot \varphi ,\Theta \bigr )$ returns unrealizable , we can be sure that the second reason applies. Then, we can even compute additional diagnostic information in the form of two Mealy machines  and  (by synthesizing some Mealy machine  and splitting it into $\mathcal {S}'$ and *F* by stripping off different outputs). The user can then try to find inputs for $\mathcal {S}'\circ F$ such that the resulting trace violates the specification. Failing to do so, the user will understand why no test strategy exists (see also [32]).

If the specification is as intended but no test strategy exists, we could use “collaborative” strategies. Among such strategies, we can choose one that requires as little collaboration from the adversary as necessary [19, 20]. In our setting, this means that we weaken the requirement that we find the fault regardless of the implementation of the system but rather require that we find it for maximal classes of implementations. This is not unusual in testing, which is typically explorative and does not make the guarantees that we attempt to give. For instance, if the specification is ${{\,\mathrm{\mathsf {G}}\,}}(r \rightarrow {{\,\mathrm{\mathsf {F}}\,}}g)$ with input *r* and output *g* and the fault model is ${{\,\mathrm{\mathsf {G}}\,}}\!{{\,\mathrm{\mathsf {F}}\,}}\lnot g$, then there is no test strategy that finds this fault for *all* implementations. Yet, an input sequence in which *r* is always $\mathsf {true}$ is a better test sequence than one in which *r* is always $\mathsf {false}$, because the former strategy will find the fault in *some* implementations, whereas the latter will not find the fault in *any* implementation. We leave the extension to collaborative strategies to future work.

*Complexity* Both ${\mathsf {synt}}_p(O, I, \psi , O',\Theta )$ and ${\mathsf {synt}}(O, I, \psi ,\Theta )$ are 2EXPTIME complete in $|\psi |$ [33], so the execution time of Algorithm 2, and consequently also Algorithm 1, are at most doubly exponential in $|\varphi | + |\kappa |$.

#### Theorem 2

For a system with inputs $I$, outputs $O$, and LTL specification $\varphi$ over $I\cup O$, if the fault kind $\kappa$ is of the form $\kappa =\psi$ or $\kappa = (o_i' \leftrightarrow \psi )$, where $\psi$ is an LTL formula over $I$ and $O$, $\textsc {SyntLtlTest}(I, O, \varphi , \kappa )$ will return a universally complete test suite with respect to the fault model $\delta ={{\,\mathrm{\mathsf {G}}\,}}(\kappa )$ if such a test suite exists.

#### Proof

Since ${{\,\mathrm{\mathsf {G}}\,}}(\kappa )$ implies $\mathsf {frq}(\kappa )$ for all $\mathsf {frq}\in \{{{\,\mathrm{\mathsf {F}}\,}}, {{\,\mathrm{\mathsf {G}}\,}}\! {{\,\mathrm{\mathsf {F}}\,}}, {{\,\mathrm{\mathsf {F}}\,}}\! {{\,\mathrm{\mathsf {G}}\,}}, {{\,\mathrm{\mathsf {G}}\,}}\}$, Theorem 1 and the guarantees of ${\mathsf {synt}}_p$ entail that the resulting test suite $\text {TS}$ is universally complete with respect to $\delta ={{\,\mathrm{\mathsf {G}}\,}}(\kappa )$ if $|\text {TS}|=|O|$, i.e., if SyntLtlTest found a strategy for every output. It remains to be shown that $|\text {TS}|=|O|$ for $\kappa =\psi$ or $\kappa = (o_i' \leftrightarrow \psi )$ if a universally complete test suite for $\delta ={{\,\mathrm{\mathsf {G}}\,}}(\kappa )$ exists: either Propositions 1 or 2 states that Eq. 6 holds with $\delta ={{\,\mathrm{\mathsf {G}}\,}}(\kappa )$. Thus, ${\mathsf {synt}}_p$ cannot return $\textsf {unrealizable}\,$ in SyntLtlIterate with $\mathsf {frq}= {{\,\mathrm{\mathsf {G}}\,}}$, so $|\text {TS}|$ must be equal to $|O|$ in this case.

Theorem 2 states that SyntLtlTest is not only sound but also complete for many interesting fault models such as stuck-at faults or permanent bit-flips. For $\kappa =\psi$, Theorem 2 can even be strengthened to hold for all $\delta =\mathsf {frq}(\kappa )$ with $\mathsf {frq}\in \{{{\,\mathrm{\mathsf {F}}\,}}, {{\,\mathrm{\mathsf {G}}\,}}\! {{\,\mathrm{\mathsf {F}}\,}}, {{\,\mathrm{\mathsf {F}}\,}}\! {{\,\mathrm{\mathsf {G}}\,}}, {{\,\mathrm{\mathsf {G}}\,}}\}$.

### Extensions and variants

A test suite computed by SyntLtlTest for specification $\varphi$ and fault model $\delta$ is universally complete and detects all faults with respect to $\varphi$ and $\delta$ independent of the implementation and the concrete fault manifestation if the fault manifests at one of the observable outputs as illustrated in Fig. 4.

In this section, we discuss some alternatives and extensions of our approach to improve fault coverage and performance.

*User-specified fault frequencies* Besides the four fault frequencies (${{\,\mathrm{\mathsf {G}}\,}}$, ${{\,\mathrm{\mathsf {F}}\,}}\!{{\,\mathrm{\mathsf {G}}\,}}$, ${{\,\mathrm{\mathsf {G}}\,}}\!{{\,\mathrm{\mathsf {F}}\,}}$, and ${{\,\mathrm{\mathsf {F}}\,}}$), other fault frequencies (with different precedences) may be of interest, e.g., if a specific time step is of special interest. Algorithm 2 supports full LTL and thus the procedure can be extended by replacing Line 2 by “**for each**$\mathsf {frq}$ from $\mathsf {Frq}$ in this order”, where $\mathsf {Frq}$ is an additional parameter provided by the user.

*Faults at inputs* In the fault model in the previous section, we only consider faults at the outputs. However, considering SUTs that behave as if they would have read a faulty input is possible as well (by changing Line 3 in Algorithm 1 to “**for each**$o\in I\cup O$**do**”).

*Multiple faults* Faults that occur simultaneously at multiple (inputs or) outputs $\{o_1, \ldots , o_k\}\subseteq O$ can be considered by computing a test strategy

$$\begin{aligned} \mathcal {T}:= {\mathsf {synt}}_p\left( O\cup \{o_1', \ldots , o_k'\}, I, (\varphi [o_1\leftarrow o_1', \ldots , o_k\leftarrow o_k'] \wedge \bigwedge _{i=1}^{k} \delta _i) \rightarrow \lnot \varphi , O,\Theta \right) , \end{aligned}$$

where the fault model $\delta _i$ can be different for different outputs $o_i \in \{o_1, \ldots , o_k\}$.

*Faults within a SUT* If a fault manifests in a *conditional fault* in a system implementation, a universally complete $\text {TS}$ may not be able to uncover the fault (see Example 2).

#### Example 2

**Fig. 7:**
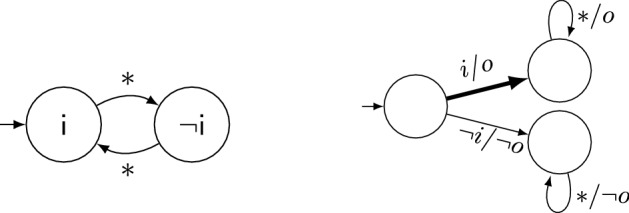
Test strategy $\mathcal {T}_6$ and a faulty system implementation of the specification $\varphi = {{\,\mathrm{\mathsf {G}}\,}}((i \leftrightarrow {{\,\mathrm{\mathsf {X}}\,}}\lnot i) \rightarrow {{\,\mathrm{\mathsf {X}}\,}}o)$

Consider a system with input $I=\{i\}$, output $O=\{o\}$, and specification $\varphi = {{\,\mathrm{\mathsf {G}}\,}}((i \leftrightarrow {{\,\mathrm{\mathsf {X}}\,}}\lnot i) \rightarrow {{\,\mathrm{\mathsf {X}}\,}}o)$. The specification enforces *o* to be set to $\mathsf {true}$ whenever input *i* alternates between $\mathsf {true}$ and $\mathsf {false}$ in consecutive time steps. Consider a stuck-at-0 fault $\delta = {{\,\mathrm{\mathsf {G}}\,}}\! {{\,\mathrm{\mathsf {F}}\,}}\lnot o$ at the output *o*. The test suite $\text {TS}= \{\mathcal {T}_6\}$ with the test strategy $\mathcal {T}_6$ illustrated in Fig. 7(on the left) is universally complete with respect to $\delta$. The test strategy $\mathcal {T}_6$ flips input *i* in every time step and thus forces the system to set $o = \mathsf {true}$ in the second time step. Now consider the concrete and faulty system implementation in Fig. 7(on the right) of $\varphi$. The test strategy $\mathcal {T}_6$, when executed, first follows the bold edge and then remains forever in the same state. As a consequence, the fault in the system implementation, i.e., *o* stuck-at-0, is not uncovered. To uncover the fault, *i* has to be set to $\mathsf {false}$ in the initial state.

Faults within a system implementation can be considered by computing more than one test strategy for a given test objective. We extend Algorithm 1 to generate a bounded number *b* of test strategies by setting $\Theta$ = $\text {TS}$ in Line 4 and enclosing the line by a **while**-loop that uses an additional integer variable *c* to count the number of test strategies generated per output $o_i$. The **while**-loop terminates if no new test strategy could be generated or if *c* becomes equal to *b*. Note that this approach is correct in the sense that all computed test strategies are universally complete with respect to the fault model $\mathsf {frq}(\kappa )$; however, in many cases it is more efficient to determine the lowest fault frequency first in Line 4 of Algorithm 2 and then generate multiple test strategies with the same (or higher) frequency by enclosing Line 3 with the **while**-loop.

*Test strategy generalization* A synthesis procedure usually assigns concrete values to all variables in every state of the generated test strategy. In many cases, however, not all assignments are necessary to enforce a test objective (see Example 3).

**Fig. 8:**
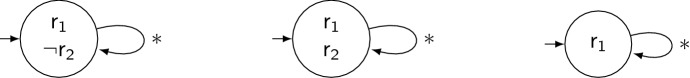
Test strategy $\mathcal {T}_7$ on the left, $\mathcal {T}_8$ in the middle and $\mathcal {T}_9$ on the right

#### Example 3

Consider a system with inputs $I=\{r_1, r_2\}$ and outputs $O=\{g_1, g_2\}$, which implements the specification of a two-input arbiter $\varphi = {{\,\mathrm{\mathsf {G}}\,}}(r_1 \rightarrow {{\,\mathrm{\mathsf {F}}\,}}g_1) \wedge {{\,\mathrm{\mathsf {G}}\,}}(r_2 \rightarrow {{\,\mathrm{\mathsf {F}}\,}}g_2) \wedge {{\,\mathrm{\mathsf {G}}\,}}( \lnot g_1 \vee \lnot g_2)$, i.e., every request $r_i$ shall eventually be granted by setting $g_i$ to $\mathsf {true}$ and there shall never be two grants at the same time. A valid test strategy $\mathcal {T}_7$ that tests for a stuck-at-0 fault of signal $g_1$ from some point in time onwards may simply set $r_1=\mathsf {true}$ and $r_2=\mathsf {false}$ all the time (see Fig. 8). This forces the system in every time step to eventually grant this one request by setting $g_1 = \mathsf {true}$. Another valid test strategy $\mathcal {T}_8$ sets $r_1=\mathsf {true}$ and $r_2=\mathsf {true}$ all the time (see Fig. 8). Now the system has to grant both requests eventually. Both $\mathcal {T}_7$ and $\mathcal {T}_8$ test for the defined stuck-at-0 fault of signal $g_1$ from some point in time onwards but will likely execute different paths in the SUT. Thus, considering the more general strategy $\mathcal {T}_9$ (see Fig. 8) that sets $r_1=\mathsf {true}$ all the time but puts no restrictions on the value of $r_2$, allows the tester to evaluate different paths in the SUT while still testing for the defined fault class.


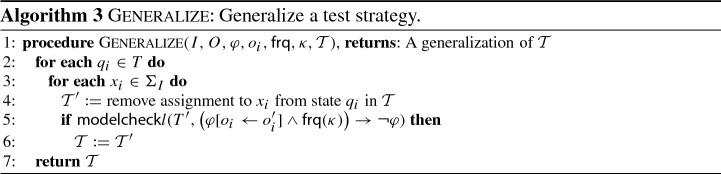


The procedure in Algorithm 3 generalizes a given test strategy $\mathcal {T}$ by systematically removing variable assignments from states and employing a model-checking procedure to ensure that the generalized test strategy still enforces the same test objective. The procedure loops in Line 2 over all states of $\mathcal {T}$ and in Line 3 over all inputs. In Line 4 the assignment to the input $x_i$ in a state is removed such that the corresponding variable becomes non-deterministic. If the resulting test strategy still enforce the test objective, then $\mathcal {T}$ is replaced by its generalization. Otherwise, the change is reverted. Algorithm 3 is integrated into Algorithm 2 and applied in Line 5 to generalize each generated test strategy.

Note that generalizing a test strategy is a special way of computing multiple concrete test strategies, which was discussed in the previous section. However, generalization may fail when computing multiple strategies succeeds (by following different paths).

*Optimization for full observability* If we restrict our perspective to the case with no partial information, i.e., all signals are fully observable, we can employ the optimization discussed in Proposition 2 to improve the performance of test strategy generation. In Line 3 of Algorithm 2 we drop a part of the assumption and simplify the synthesis step to $\mathcal {T}_i := {\mathsf {synt}}\bigl (O, I, \mathsf {frq}(\kappa ) \rightarrow \lnot \varphi , \Theta \bigr )$ for cases in which $\kappa$ does not refer to a hidden signal $o_i'$. Also, for a fault model $\delta$ that describes a fault of kind $\kappa = (o_i' \leftrightarrow \psi )$, where $\psi$ is an LTL formula over $I$ and $O$, we can drop the part of the assumption according to Proposition 1 if $\mathsf {frq}={{\,\mathrm{\mathsf {G}}\,}}$. This simplifies Line 3 of Algorithm 2 to $\mathcal {T}_i := {\mathsf {synt}}\bigl (O, I, \varphi [o_i\leftarrow \psi ] \rightarrow \lnot \varphi ,\Theta \bigr )$. These simplifications, moreover, no longer require a synthesis procedure with partial information and thus, a larger set of synthesis tools is supported.

*Other specification formalisms* We worked out our approach for LTL, but it works for other languages if (1) the language is closed under Boolean connectives $(\wedge , \lnot )$, (2) the desired fault models are expressible, and (3) a synthesis procedure (with partial information) is available. These prerequisites do not only apply to many temporal logics but also to various kinds of automata over infinite words.

## Case study

To evaluate our approach, we apply it in a case study on a real component of a satellite system that is currently under development. We first present the system under test and specify a version of the respective component in LTL. Using this specification, we compute a set of test strategies and evaluate them on a real implementation. Additional case studies can be found in [10].

### Eu:CROPIS FDIR specification

An important task of each space and satellite system is to maintain its health state and react on failure. In modern space systems, this task is encapsulated in the *Fault Detection, Isolation, and Recovery* (FDIR) component, which collects information from all relevant sensors and on-board computers, analyzes and assesses the data in terms of correctness and health, and initiates recovery actions if necessary. The FDIR component is organized hierarchically in multiple levels [51] with the overall objective of maximizing the life-time and correct operation of the system.

In this section, we focus on system-level FDIR and present a high-level abstraction of a part of the FDIR mechanisms used in the Eu:CROPIS satellite mission as a case study for adaptive test strategy generation. On the system-level, the FDIR mechanism deals with coarse-grained anomalies of the system behavior such as erroneous sensor data or impossible combinations of signals. Likewise the recovery actions are limited to restarting certain sub-systems, switching between redundant sub-systems if available, or switching into the satellite’s safe mode. The FDIR component is highly safety- and mission-critical. If recovery on this level fails, in many cases the mission has to be considered lost.

**Fig. 9:**
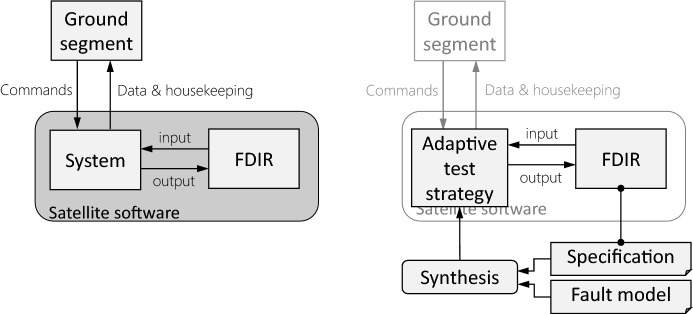
FDIR in practice (left) and the intended test setup (right)

**Fig. 10:**
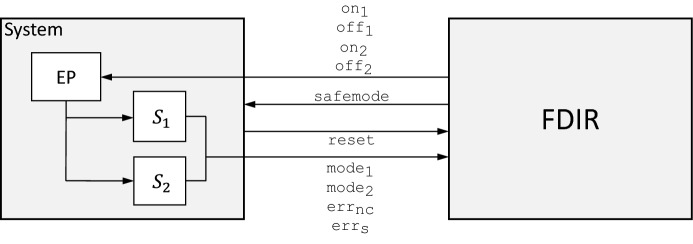
High-level overview of the satellite software of Eu:CROPIS

*Eu:CROPIS FDIR* In Fig. 9 we illustrate where the FDIR component for the magnetic torquers of the Eu:CROPIS on-board computing system is placed in practice and in Fig. 10, we give a high-level overview of the FDIR component and its environment. The FDIR component regularly obtains housekeeping information from two redundantly-designed control units, $S_1$ and $S_2$, which control the magnetic torquers of the satellite, and interacts with them via the electronic power system, EP. The control units $S_1$ and $S_2$ have the same functionality, but only one of them is active at any time. The other control unit serves as a backup that can be activated if necessary. The FDIR component signals the activation (or deactivation) of a control unit to the EP which regulates the power supply.

We distinguish two types of errors, called *non-critical error* and *severe error*, signaled to the FDIR component via housekeeping information. In case of a non-critical error, two recovery actions are possible. Either the erroneous control unit is disabled for a short time and enabled afterwards again or the erroneous control unit is disabled and the redundant control unit is activated to take over its task. In case of the severe error, however, only the latter recovery action is allowed, i.e., the erroneous control unit has to be disabled and the redundant control unit has to be activated. If this happens more than once and the redundant control unit as well shows erroneous behavior, the FDIR component initiates a switch of the satellite mode into safe mode. The safe mode is a fall-back satellite mode designed to give the operators on ground the maximum amount of time to analyze and fix the problem. It is only invoked once a problem cannot be solved on-board and requires input from the operators to restore nominal operations.

*LTL specification* We model the specification of the FDIR component in LTL. Let $I_{FDIR}$ = {$\mathtt{mode}_{\mathtt{1}}\,$, $\mathtt{mode}_{\mathtt{2}}\,$, $\mathtt{err}_{\mathtt{nc}}\,$, $\mathtt{err}_{\mathtt{s}}\,$, $\mathtt{reset}\,$} and $O_{FDIR}$ = {$\mathtt{on}_{\mathtt{1}}\,$, $\mathtt{off}_{\mathtt{1}}\,$, $\mathtt{on}_{\mathtt{2}}\,$, $\mathtt{off}_{\mathtt{2}}\,$, $\mathtt{safemode}\,$} be the Boolean variables corresponding to the input signals and the output signals of the FDIR component, respectively.

These Boolean variables are abstractions of the real hardware/software implementation. The values of the Boolean variables are automatically extracted from the housekeeping information which is periodically collected from EP ($\mathtt{mode}_{\mathtt{1}}\,$, $\mathtt{mode}_{\mathtt{2}}\,$) and $S_1$ or $S_2$ ($\mathtt{err}_{\mathtt{nc}}\,$, $\mathtt{err}_{\mathtt{s}}\,$). The two error variables encompass multiple error conditions (e.g. communication timeouts, invalid responses, electrical errors like over-current or under-voltage, etc.) which are detected by the sub-system. The $\mathtt{reset}\,$ variable corresponds to a telecommand sent from ground to the FDIR component. For the output direction the values of the variables are used to generate commands which are sent to the EP or the satellite mode handling component. Additionally, we use the auxiliary Boolean variables $O^\prime$ = {$\mathtt{lastup}\,$, $\mathtt{allowswitch}\,$} to model state information on specification level. These auxiliary variables do not correspond to real signals in the system, but are used as unobservable outputs of the FDIR component. In Table 1, we present a summary of the Boolean variables involved in the specification and describe their meaning.

The LTL specification of the FDIR component is of form

$$\begin{aligned} \left( \bigwedge _{i=1}^{6} A_i \right) \rightarrow \left( \bigwedge _{i=1}^{13} G_i \right) \end{aligned}$$

and consists of the six assumptions $A_{1}$–$A_{6}$ and the thirteen guarantees $G_{1}$–$G_{13}$. All properties are listed in Table 2, expressing the following intentions:

**Table 1:** Descriptions of inputs and outputs of the FDIR component

Boolean variable	Description
$\mathtt{mode}_{\mathtt{1}}\,$	$\mathsf {true}$ iff $S_1$ is activated
$\mathtt{mode}_{\mathtt{2}}\,$	$\mathsf {true}$ iff $S_2$ is activated
$\mathtt{err}_{\mathtt{nc}}\,$	$\mathsf {true}$ iff a non-critical error is signaled by $S_1$ or $S_2$
$\mathtt{err}_{\mathtt{s}}\,$	$\mathsf {true}$ iff a severe error is signaled by $S_1$ or $S_2$
reset	$\mathsf {true}$ iff the FDIR component is reset
$\mathtt{on}_{\mathtt{1}}\,$	$\mathsf {true}$ iff $S_1$ shall be switched on
$\mathtt{off}_{\mathtt{1}}\,$	$\mathsf {true}$ iff $S_1$ shall be switched off
$\mathtt{on}_{\mathtt{2}}\,$	$\mathsf {true}$ iff $S_2$ shall be switched on
$\mathtt{off}_{\mathtt{2}}\,$	$\mathsf {true}$ iff $S_2$ shall be switched off
safemode	$\mathsf {true}$ iff the FDIR component initiates the safemode of the satellite
lastup	$\mathsf {true}$ if the last active system was $S_1$ and $\mathsf {false}$ if the last active system was $S_2$
allowswitch	$\mathsf {true}$ iff a switch of $S_1$ to $S_2$ or $S_2$ to $S_1$ is allowed

**Table 2:** Temporal specification of system-level FDIR component in LTL

**Assumptions**$A_{1}$–$A_{6}$	
$A_1$	${{\,\mathrm{\mathsf {G}}\,}}((\lnot \mathtt{mode}_{\mathtt{2}}\,\wedge \lnot \mathtt{mode}_{\mathtt{1}}\,) \rightarrow \lnot \mathtt{err}_{\mathtt{nc}}\,\wedge \lnot \mathtt{err}_{\mathtt{s}}\,)$
$A_2$	${{\,\mathrm{\mathsf {G}}\,}}(\lnot \mathtt{err}_{\mathtt{nc}}\,\vee \lnot \mathtt{err}_{\mathtt{s}}\,) \wedge {{\,\mathrm{\mathsf {G}}\,}}(\mathtt{reset}\,\rightarrow \lnot \mathtt{err}_{\mathtt{nc}}\,\wedge \lnot \mathtt{err}_{\mathtt{s}}\,)$
$A_3$	${{\,\mathrm{\mathsf {G}}\,}}(\mathtt{reset}\,\rightarrow {{\,\mathrm{\mathsf {X}}\,}}(\mathtt{mode}_{\mathtt{2}}\,\oplus \mathtt{mode}_{\mathtt{1}}\,))$
$A_4$	$$\begin{aligned} {{\,\mathrm{\mathsf {G}}\,}}(&\lnot \mathtt{mode}_{\mathtt{1}}\,\wedge \mathtt{on}_{\mathtt{1}}\,\wedge \lnot \mathtt{off}_{\mathtt{1}}\,\wedge \lnot \mathtt{on}_{\mathtt{2}}\,\wedge \lnot \mathtt{off}_{\mathtt{2}}\,\wedge \lnot \mathtt{reset}\,\wedge \lnot \mathtt{safemode}\,) \rightarrow \\ \quad&{{\,\mathrm{\mathsf {X}}\,}}\mathtt{mode}_{\mathtt{1}}\,\wedge (\mathtt{mode}_{\mathtt{2}}\,\leftrightarrow {{\,\mathrm{\mathsf {X}}\,}}\mathtt{mode}_{\mathtt{2}}\,)) \end{aligned}$$
	$$\begin{aligned} {{\,\mathrm{\mathsf {G}}\,}}(&\lnot \mathtt{mode}_{\mathtt{2}}\,\wedge \lnot \mathtt{on}_{\mathtt{1}}\,\wedge \lnot \mathtt{off}_{\mathtt{1}}\,\wedge \mathtt{on}_{\mathtt{2}}\,\wedge \lnot \mathtt{off}_{\mathtt{2}}\,\wedge \lnot \mathtt{reset}\,\wedge \lnot \mathtt{safemode}\,\rightarrow \\&{{\,\mathrm{\mathsf {X}}\,}}\mathtt{mode}_{\mathtt{2}}\,\wedge (\mathtt{mode}_{\mathtt{1}}\,\leftrightarrow {{\,\mathrm{\mathsf {X}}\,}}\mathtt{mode}_{\mathtt{1}}\,)) \end{aligned}$$
$A_5$	$$\begin{aligned} {{\,\mathrm{\mathsf {G}}\,}}(&\mathtt{mode}_{\mathtt{1}}\,\wedge \lnot \mathtt{on}_{\mathtt{1}}\,\wedge \mathtt{off}_{\mathtt{1}}\,\wedge \lnot \mathtt{on}_{\mathtt{2}}\,\wedge \lnot \mathtt{off}_{\mathtt{2}}\,\wedge \lnot \mathtt{reset}\,\wedge \lnot \mathtt{safemode}\,\rightarrow \\&{{\,\mathrm{\mathsf {X}}\,}}\lnot \mathtt{mode}_{\mathtt{1}}\,\wedge (\mathtt{mode}_{\mathtt{2}}\,\leftrightarrow {{\,\mathrm{\mathsf {X}}\,}}\mathtt{mode}_{\mathtt{2}}\,) ) \end{aligned}$$
	$$\begin{aligned} {{\,\mathrm{\mathsf {G}}\,}}(&\mathtt{mode}_{\mathtt{2}}\,\wedge \lnot \mathtt{on}_{\mathtt{1}}\,\wedge \lnot \mathtt{off}_{\mathtt{1}}\,\wedge \lnot \mathtt{on}_{\mathtt{2}}\,\wedge \mathtt{off}_{\mathtt{2}}\,\wedge \lnot \mathtt{reset}\,\wedge \lnot \mathtt{safemode}\,\rightarrow \\&{{\,\mathrm{\mathsf {X}}\,}}\lnot \mathtt{mode}_{\mathtt{2}}\,\wedge (\mathtt{mode}_{\mathtt{1}}\,\leftrightarrow {{\,\mathrm{\mathsf {X}}\,}}\mathtt{mode}_{\mathtt{1}}\,) ) \end{aligned}$$
$A_6$	$$\begin{aligned} {{\,\mathrm{\mathsf {G}}\,}}(&(\lnot (\lnot \mathtt{on}_{\mathtt{2}}\,\wedge \lnot \mathtt{off}_{\mathtt{1}}\,\wedge \lnot \mathtt{on}_{\mathtt{1}}\,\wedge \lnot \mathtt{off}_{\mathtt{2}}\,) \wedge {{\,\mathrm{\mathsf {X}}\,}}(\lnot \mathtt{on}_{\mathtt{2}}\,\wedge \lnot \mathtt{off}_{\mathtt{1}}\,\wedge \lnot \mathtt{on}_{\mathtt{1}}\,\wedge \lnot \mathtt{off}_{\mathtt{2}}\,) \wedge \\&(\lnot \mathtt{reset}\,\wedge {{\,\mathrm{\mathsf {X}}\,}}\lnot \mathtt{reset}\,\wedge \lnot \mathtt{safemode}\,\wedge {{\,\mathrm{\mathsf {X}}\,}}\lnot \mathtt{safemode}\,) \rightarrow \\&{{\,\mathrm{\mathsf {X}}\,}}((\mathtt{mode}_{\mathtt{2}}\,\leftrightarrow {{\,\mathrm{\mathsf {X}}\,}}\mathtt{mode}_{\mathtt{2}}\,) \wedge (\mathtt{mode}_{\mathtt{1}}\,\leftrightarrow {{\,\mathrm{\mathsf {X}}\,}}\mathtt{mode}_{\mathtt{1}}\,) ) \end{aligned}$$
**Guarantees**$G_{1}$–$G_{13}$	
$G_1$	${{\,\mathrm{\mathsf {G}}\,}}((\mathtt{on}_{\mathtt{1}}\,\wedge \lnot \mathtt{on}_{\mathtt{2}}\,) \rightarrow ({{\,\mathrm{\mathsf {X}}\,}}\mathtt{lastup}\,))$
	$${{\,\mathrm{\mathsf {G}}\,}}((\lnot \mathtt{on}_{\mathtt{1}}\,\wedge \mathtt{on}_{\mathtt{2}}\,) \rightarrow ({{\,\mathrm{\mathsf {X}}\,}}\lnot \mathtt{lastup}\,))$$
	$${{\,\mathrm{\mathsf {G}}\,}}( (\lnot \mathtt{on}_{\mathtt{1}}\,\wedge \lnot \mathtt{on}_{\mathtt{2}}\,) \rightarrow (\mathtt{lastup}\,\leftrightarrow {{\,\mathrm{\mathsf {X}}\,}}\mathtt{lastup}\,))$$
$G_2$	${{\,\mathrm{\mathsf {G}}\,}}(\mathtt{on}_{\mathtt{1}}\,\rightarrow \lnot \mathtt{off}_{\mathtt{1}}\,\wedge \lnot \mathtt{on}_{\mathtt{2}}\,\wedge \lnot \mathtt{off}_{\mathtt{2}}\,)$
	$${{\,\mathrm{\mathsf {G}}\,}}(\mathtt{off}_{\mathtt{1}}\,\rightarrow \lnot \mathtt{on}_{\mathtt{1}}\,\wedge \lnot \mathtt{on}_{\mathtt{2}}\,\wedge \lnot \mathtt{off}_{\mathtt{2}}\,)$$
	$${{\,\mathrm{\mathsf {G}}\,}}(\mathtt{on}_{\mathtt{2}}\,\rightarrow \lnot \mathtt{on}_{\mathtt{1}}\,\wedge \lnot \mathtt{off}_{\mathtt{1}}\,\wedge \lnot \mathtt{off}_{\mathtt{2}}\,)$$
	$${{\,\mathrm{\mathsf {G}}\,}}(\mathtt{off}_{\mathtt{2}}\,\rightarrow \lnot \mathtt{on}_{\mathtt{1}}\,\wedge \lnot \mathtt{on}_{\mathtt{2}}\,\wedge \lnot \mathtt{off}_{\mathtt{1}}\,)$$
$G_3$	${{\,\mathrm{\mathsf {G}}\,}}(\lnot \mathtt{mode}_{\mathtt{2}}\,\wedge \lnot \mathtt{mode}_{\mathtt{1}}\,\rightarrow {{\,\mathrm{\mathsf {F}}\,}}(\mathtt{reset}\,\vee \mathtt{on}_{\mathtt{2}}\,\vee \mathtt{on}_{\mathtt{1}}\,\vee \mathtt{safemode}\,))$
$G_4$	${{\,\mathrm{\mathsf {G}}\,}}(\mathtt{allowswitch}\,\rightarrow \lnot \mathtt{safemode}\,)$
$G_5$	${{\,\mathrm{\mathsf {G}}\,}}((\mathtt{mode}_{\mathtt{2}}\,\vee \mathtt{mode}_{\mathtt{1}}\,) \rightarrow \lnot \mathtt{on}_{\mathtt{1}}\,\wedge \lnot \mathtt{on}_{\mathtt{2}}\,)$
$G_6$	${{\,\mathrm{\mathsf {G}}\,}}(\lnot \mathtt{allowswitch}\,\wedge \mathtt{lastup}\,\rightarrow \lnot \mathtt{on}_{\mathtt{2}}\,)$
	$${{\,\mathrm{\mathsf {G}}\,}}(\lnot \mathtt{allowswitch}\,\wedge \lnot \mathtt{lastup}\,\rightarrow \lnot \mathtt{on}_{\mathtt{1}}\,)$$
$G_7$	${{\,\mathrm{\mathsf {G}}\,}}(\lnot \mathtt{reset}\,\wedge \mathtt{allowswitch}\,\wedge \mathtt{lastup}\,\wedge \mathtt{on}_{\mathtt{2}}\,\rightarrow {{\,\mathrm{\mathsf {X}}\,}}\lnot \mathtt{allowswitch}\,)$
	$${{\,\mathrm{\mathsf {G}}\,}}(\lnot \mathtt{reset}\,\wedge \mathtt{allowswitch}\,\wedge \lnot \mathtt{lastup}\,\wedge \mathtt{on}_{\mathtt{1}}\,\rightarrow {{\,\mathrm{\mathsf {X}}\,}}\lnot \mathtt{allowswitch}\,)$$
$G_8$	${{\,\mathrm{\mathsf {G}}\,}}((\mathtt{allowswitch}\,\wedge \lnot (((\mathtt{lastup}\,\wedge \mathtt{on}_{\mathtt{2}}\,) \vee (\lnot \mathtt{lastup}\,\wedge \mathtt{on}_{\mathtt{1}}\,)))) \rightarrow {{\,\mathrm{\mathsf {X}}\,}}\mathtt{allowswitch}\,)$
$G_9$	${{\,\mathrm{\mathsf {G}}\,}}(\mathtt{reset}\,\rightarrow {{\,\mathrm{\mathsf {X}}\,}}\mathtt{allowswitch}\,)$
$G_{10}$	${{\,\mathrm{\mathsf {G}}\,}}(\mathtt{safemode}\,\rightarrow (\lnot \mathtt{on}_{\mathtt{1}}\,\wedge \lnot \mathtt{on}_{\mathtt{2}}\,))$
$G_{11}$	${{\,\mathrm{\mathsf {G}}\,}}( \lnot \mathtt{allowswitch}\,\wedge \lnot \mathtt{reset}\,\rightarrow {{\,\mathrm{\mathsf {X}}\,}}\lnot \mathtt{allowswitch}\,)$
$G_{12}$	$$ \begin{aligned} {{\,\mathrm{\mathsf {G}}\,}}(&(\mathtt{err}_{\mathtt{s}}\,\wedge \mathtt{mode}_{\mathtt{1}}\,\wedge \lnot \mathtt{reset}\,) \rightarrow \\&{{\,\mathrm{\mathsf {F}}\,}}(\mathtt{reset}\,\vee \mathtt{safemode}\,\vee \mathtt{mode}_{\mathtt{2}}\,\vee (\mathtt{mode}_{\mathtt{1}}\,\mathbin {\mathsf {U}}(\mathtt{mode}_{\mathtt{1}}\,\wedge \lnot \mathtt{err}_{\mathtt{s}}\,)))) \end{aligned}$$
	$$\begin{aligned} {{\,\mathrm{\mathsf {G}}\,}}(&(\mathtt{err}_{\mathtt{s}}\,\wedge \mathtt{mode}_{\mathtt{2}}\,\wedge \lnot \mathtt{reset}\,) \rightarrow \\&{{\,\mathrm{\mathsf {F}}\,}}(\mathtt{reset}\,\vee \mathtt{safemode}\,\vee \mathtt{mode}_{\mathtt{1}}\,\vee (\mathtt{mode}_{\mathtt{2}}\,\mathbin {\mathsf {U}}(\mathtt{mode}_{\mathtt{2}}\,\wedge \lnot \mathtt{err}_{\mathtt{s}}\,)))) \end{aligned}$$
$G_{13}$	${{\,\mathrm{\mathsf {G}}\,}}((\mathtt{err}_{\mathtt{nc}}\,\wedge \mathtt{mode}_{\mathtt{1}}\,\wedge \lnot \mathtt{reset}\,) \rightarrow {{\,\mathrm{\mathsf {F}}\,}}(\mathtt{reset}\,\vee \mathtt{safemode}\,\vee \mathtt{mode}_{\mathtt{2}}\,\vee (\mathtt{mode}_{\mathtt{1}}\,\wedge \lnot \mathtt{err}_{\mathtt{nc}}\,)))$
	$${{\,\mathrm{\mathsf {G}}\,}}((\mathtt{err}_{\mathtt{nc}}\,\wedge \mathtt{mode}_{\mathtt{2}}\,\wedge \lnot \mathtt{reset}\,) \rightarrow {{\,\mathrm{\mathsf {F}}\,}}(\mathtt{reset}\,\vee \mathtt{safemode}\,\vee \mathtt{mode}_{\mathtt{1}}\,\vee (\mathtt{mode}_{\mathtt{2}}\,\wedge \lnot \mathtt{err}_{\mathtt{nc}}\,)))$$

$$A_1$$
Whenever both systems are off, then there is no running system that can have an error. Thus, the error signals have to be low as well.
$$A_2$$
The error signals are mutual exclusive. If the environment enforces a reset then both error signals have to be low, because we assume that ground control has taken care of the errors.
$$A_3$$
After a reset enforced by the environment, one of the two systems has to be running and the other has to be off.
$$A_4$$
Whenever the FDIR component sends $\mathtt{on}_{\mathtt{1}}\,$, we assume that in the next time step system number one is running ($\mathtt{mode}_{\mathtt{1}}\,$) and the state of the second system ($\mathtt{mode}_{\mathtt{2}}\,$) does not change. The same assumption applies analogously for $\mathtt{on}_{\mathtt{2}}\,$.
$$A_5$$
Whenever the FDIR component sends $\mathtt{off}_{\mathtt{1}}\,$, we assume that in the next time step system number one is off ($\lnot \mathtt{mode}_{\mathtt{1}}\,$) and the state of the second system ($\mathtt{mode}_{\mathtt{2}}\,$) does not change. The same assumption applies analogously for $\mathtt{off}_{\mathtt{2}}\,$.
$$A_6$$
We assume that the environment, more specifically the electronic power unit, is not immediately free to change the state of the systems when there is no message from the FDIR component. It has to wait for one more time step (with no messages of the FDIR component).
$$G_1$$
This guarantee stores which system was last activated by the FDIR component.
$$G_2$$
We require the signals $\mathtt{on}_{\mathtt{1}}\,$, $\mathtt{off}_{\mathtt{1}}\,$, $\mathtt{on}_{\mathtt{2}}\,$ and $\mathtt{off}_{\mathtt{2}}\,$ to be mutually exclusively set to high.
$$G_3$$
Whenever both systems are off, then the FDIR component eventually requests to switch on one of the systems ($\mathtt{on}_{\mathtt{1}}\,$, $\mathtt{on}_{\mathtt{2}}\,$) or activates $\mathtt{safemode}\,$ or observes a $\mathtt{reset}\,$.
$$G_4$$
We restrict the FDIR component to not enter $\mathtt{safemode}\,$ as long as the component can switch to the backup system.
$$G_5$$
The FDIR component must not request to switch on one of the systems ($\mathtt{on}_{\mathtt{1}}\,$, $\mathtt{on}_{\mathtt{2}}\,$) as long as one of the systems is running.
$$G_6$$
Whenever the FDIR component is not allowed anymore to switch to the backup system, then it must not request to switch the backup system on.
$$G_7$$
Once the FDIR component switches to the backup system it is not allowed anymore to switch again (unless the environment performs a reset, see G9).
$$G_8$$
As long as the FDIR component only restarts the same system it is still allowed to switch in the future.
$$G_9$$
A $\mathtt{reset}\,$ by the environment allows the FDIR component again to switch to the backup system if required.
$$G_{10}$$
Whenever the FDIR component is in $\mathtt{safemode}\,$ it must not request to switch-on one of the systems ($\mathtt{on}_{\mathtt{1}}\,$,$\mathtt{on}_{\mathtt{2}}\,$).
$$G_{11}$$
Once a switch is not allowed anymore and the environment does not perform a reset, then the switch is also not allowed in the next time step.
$$G_{12}$$
Whenever the FDIR component observes a server error ($\mathtt{err}_{\mathtt{s}}\,$), it must eventually switch to the backup system or activate $\mathtt{safemode}\,$ unless the environment performs a $\mathtt{reset}\,$ or the error disappears by itself (without restarting the system).
$$G_{13}$$
Whenever the FDIR component observes a non-critical error ($\mathtt{err}_{\mathtt{nc}}\,$), it must eventually switch to the backup system or activate $\mathtt{safemode}\,$ or the error disappears (restarting the currently running system is allowed).

### Experimental results

In this section, we present experimental results for generating test strategies for the LTL specification of the Eu:CROPIS FDIR component. We first analyze runtime and memory consumption of test strategy synthesis, and then evaluate the effectiveness of the generated test strategies on a concrete implementation of the FDIR component. The proposed test strategy synthesis approach, however, is a black-box testing technique, independent of the concrete implementation and can be applied even if no implementation is available. The synthesized test strategies do not contain the test oracle; for the experiments, we use a concrete implementation, that was manually verified, as test oracle.

#### Test strategy computation

*Experimental setting* All experiments for computing test strategies are conducted in a virtual machine with a 64 bit Linux system using a single core of an Intel i5 CPU running at 2.60 GHz. We use the synthesis procedure PARTY [31] as black-box, which implements SMT-based bounded synthesis for full LTL and, thus, we call our tool PARTYStrategy.^6^

*Test strategy computation* From the previously described LTL specification, we compute test strategies for the outputs $\mathtt{on}_{\mathtt{1}}\,$, $\mathtt{off}_{\mathtt{1}}\,$ and safemode of the FDIR component considering the fault models stuck-at-0, stuck-at-1, and bit-flip with the lowest possible fault frequencies. These are general fault assumptions and cover faults where the specification is violated with this signal being high (stuck-at-1), faults where the specification is violated with this signal being low (stuck-at-0) and faults where the specification is violated with this signal having the wrong polarity (bit-flip). We do not synthesize test strategies for the outputs $\mathtt{on}_{\mathtt{2}}\,$ and $\mathtt{off}_{\mathtt{2}}\,$ because they behave identical to $\mathtt{on}_{\mathtt{1}}\,$ and $\mathtt{off}_{\mathtt{1}}\,$, respectively, if the role of $S_1$ and $S_2$ are mutually interchanged. For synthesizing test strategies, both, the bound for the maximal number of states of a test strategy and the bound for the maximal number of test strategies, are set to four. We chose the bound to be four, because for this bound there exist strategies for all our chosen fault models and output signals. The size for the maximum number of strategies per variable and fault model is set arbitrarily to four and could also be set to a different value.

**Table 3:** Results for the FDIR specification. The suffix “k” multiplies by $10^3$

Fault	$o_i$	$\mathsf {frq}$	$|\mathcal {T}|$	Time	Peak memory
				(s)	(MB)
S-a-0	$\mathtt{on}_{\mathtt{1}}\,$	${{\,\mathrm{\mathsf {F}}\,}}\! {{\,\mathrm{\mathsf {G}}\,}}$	4	1.2k	400
	$\mathtt{off}_{\mathtt{1}}\,$	${{\,\mathrm{\mathsf {F}}\,}}\! {{\,\mathrm{\mathsf {G}}\,}}$	3	517	396
	safemode	${{\,\mathrm{\mathsf {F}}\,}}\! {{\,\mathrm{\mathsf {G}}\,}}$	4	934	324
S-a-1	$\mathtt{on}_{\mathtt{1}}\,$	${{\,\mathrm{\mathsf {G}}\,}}\!{{\,\mathrm{\mathsf {F}}\,}}$	4	438	222
	$\mathtt{off}_{\mathtt{1}}\,$	${{\,\mathrm{\mathsf {F}}\,}}\! {{\,\mathrm{\mathsf {G}}\,}}$	4	753	378
	safemode	${{\,\mathrm{\mathsf {G}}\,}}\!{{\,\mathrm{\mathsf {F}}\,}}$	3	169	192
Bit-Flip	$\mathtt{on}_{\mathtt{1}}\,$	${{\,\mathrm{\mathsf {G}}\,}}\!{{\,\mathrm{\mathsf {F}}\,}}$	4	26k	3.6k
	$\mathtt{off}_{\mathtt{1}}\,$	${{\,\mathrm{\mathsf {F}}\,}}\! {{\,\mathrm{\mathsf {G}}\,}}$	4	98.9k	4.3k
	safemode	${{\,\mathrm{\mathsf {G}}\,}}\!{{\,\mathrm{\mathsf {F}}\,}}$	3	13.1k	4.3k

In Table 3, we list the time and memory consumption for synthesizing the test strategies with our synthesis tool PARTYStrategy. The more freedom there is for implementations of the specification, the harder it becomes to compute a strategy. The search for strategies that are capable of detecting a bit-flip is the most difficult one as we cannot make use of our optimization for full observability of the output signals. For all signals with a stuck-at-0 fault and for the $\mathtt{off}_{\mathtt{1}}\,$ signal with one of the other two faults we are able to derive test strategies that can detect the fault if it is permanent from some point onwards. For the signals $\mathtt{on}_{\mathtt{1}}\,$ and safemode we are able to derive strategies for stuck-at-1 faults and bit-flips also at a lower frequency, i.e., we can detect those faults also if they occur at least infinitely often.

**Fig. 11:**
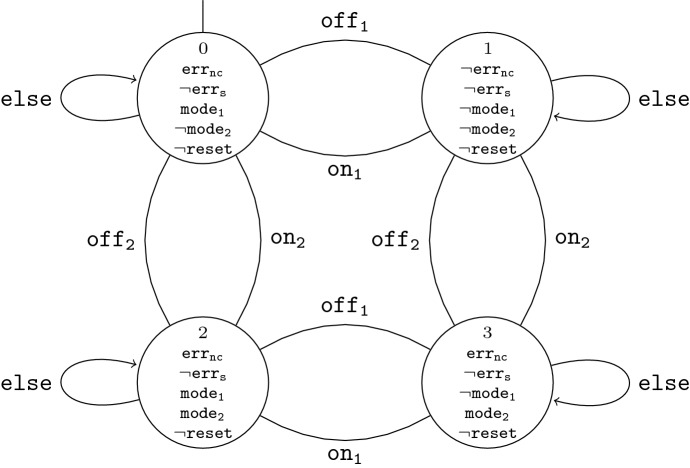
Test strategy that tests for a stuck-at-0 fault of signal $\mathtt{safemode}\,$

*Illustration of a computed strategy* We illustrate and explain one derived strategy in detail. The strategy derived for the signal safemode being stuck at 0 computed consists of four states. Figure 11 illustrates the strategy. In the first state (state 0) we have the first system running ($\mathtt{mode}_{\mathtt{1}}\,$) and set the $\mathtt{err}_{\mathtt{nc}}\,$ flag, i.e., we raise a non-critical error that requires the component to restart until the error is gone or to switch to the other system. We loop in this state until the FDIR component, if it behaves according to the specification, switches off the running system. In the next state (state 1), we do not set any input and wait for the FDIR component to eventually switch on one of the systems. If the component switches on the same system, then we go back to the previous state (state 0), if it switches on the other system we go into the next state (state 3). In this state we have the second system running ($\mathtt{mode}_{\mathtt{2}}\,$) and set again the $\mathtt{err}_{\mathtt{nc}}\,$ flag, i.e., we again raise a non-critical error. We loop in this state until the FDIR component reacts and, if it conforms to the specification, switches off the running system. Continuing according to the strategy we always raise a non-critical error whatever system the FDIR component activates. Eventually the FDIR component has to activate safemode or violate the specification. State 2 is only entered when the FDIR violates G5. In this state, it is irrelevant how the test strategy behaves because the specification has already been violated (which is easy to detect during test execution).

**Fig. 12:**
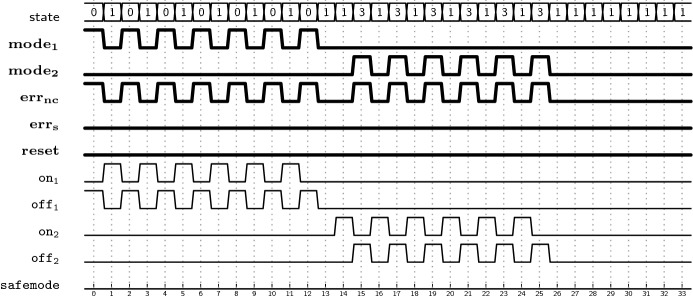
Execution trace from a faulty system under the strategy that tests for a stuck-at-0 fault of signal $\mathtt{safemode}\,$. Bold signals are controlled by the strategy

#### Test strategy evaluation

*Test setting* In the Eu:CROPIS satellite the FDIR component is implemented in software in the programming language C++. The implementation for the magnetic torquer FDIR handling is not an exact realization of the specification in Table 2 but extends it by allowing commands to the EP to be lost (e.g. due to electrical faults). This is accommodated by adding timeouts for the execution of the switch-on/off commands and reissuing the commands if the timeout is triggered.

The implementation is designed with testability and portability in mind and uses an abstract interface to access other sub-systems of the satellite. This allows engineers to exchange the used interface with a set of test adapters which connect to the signals generated by the test strategies. As we are only interested in the functional properties of the implementation, we can run the code on a normal Linux system, instead of the microprocessor which is used in the satellite. This gives access to all Linux based debugging and testing tools and allows us to use gcov to measure the line and branch coverage of the source code.

A time step of a test run consists of the following operations: request values for the input variables $I_{FDIR}$ from the test strategy; feed the values to the test adapter from which they are read by the FDIR implementation; run the FDIR implementation for one cycle; extract the output values $O_{FDIR}$ from the test adapter and feed them back to the test strategy to get new input values. For each time step, the execution trace—the values assigned to the inputs $I_{FDIR}$ and outputs $O_{FDIR}$ of the FDIR component—is recorded.

*Mutation testing* Besides line and branch coverage, we apply mutation analysis to assess the effectiveness, i.e., fault finding abilities, of a test suite. A test suite *kills* a mutant program *M* if it contains at least one test strategy that, when executed on *M* and the original program *P*, produces a trace where at least one output of *M* differs in at least one time step from the respective output of *P* (for the same input sequence). A mutant program *M* is *equivalent* to the original program *P* if *M* does not violate the specification. For our evaluation we manually identify and remove equivalent mutants.

We generate mutant programs of the FDIR component by systematically applying the following four mutations to each line of the C++ implementation:

1. Deletion of the line,
2. Replacement of true with false or false with true,
3. Replacement of == with != or != with ==, and
4. Replacement of && with || or || with &&

In total, 210 mutant programs are generated. Each having exactly one mutation. We use the GNU compiler gcc to remove all mutant programs, which do not compile and do not conform to the C++ programming language. We analyzed the remaining 105 mutants manually and identified 5 equivalent mutant programs, i.e., they do not violate the specification. We further correct this number by removing another 17 mutant programs related to un-specified implementation-specific behavior. Next, we executed all test strategies on the mutant programs for 80 time steps each and log the corresponding execution traces.

In Fig. 12 we illustrate the execution of the test strategy from Fig. 11 on a mutant program. This particular strategy aims at revealing a stuck-at-0 fault for signal $\mathtt{safemode}\,$. The test strategy first forces the FDIR component to eventually switch to the backup system. The switch happens in time step 14 after several restarts of the system. Then the strategy forces the FDIR component to eventually activate $\mathtt{safemode}\,$. However, this mutant program is faulty and instead of activating $\mathtt{safemode}\,$ the system remains silent from time step 26 onwards. Thus, violating guarantee G3.^7^

**Table 4:** Mutation coverage by fault models and signal when executing all four derived strategies

Output	Fault model			
	**S-a-0**	**S-a-1**	**Bit-flip**	All
	(%)	(%)	(%)	(%)
$\mathtt{on}_{\mathtt{1}}\,$	67.47	53.01	8.43	75.90
$\mathtt{off}_{\mathtt{1}}\,$	13.25	3.61	16.87	16.87
safemode	61.45	13.25	13.25	16.87
All	71.08	55.42	16.87	78.31

From the 83 mutant programs that violate the specification, the synthesized adaptive test strategies are able to kill 65 (78.31%). Since these test strategies are derived from requirements, without any implementation-specific knowledge, they are applicable to any system that claims to implement the specification. The mutation score of $78.31\%$ motivate that the synthesized adaptive test strategies—although computed only for simple specific fault models—are sensitive to other faults.

In Table 4, we present the mutation scores for the three signals $\mathtt{on}_{\mathtt{1}}\,$, $\mathtt{off}_{\mathtt{1}}\,$, and safemode and the three fault models stuck-at-0 (**S-a-0**), stuck-at-1 (**S-a-1**), and bit-flip (**Bit-flip**). The last column and last row show the mutation scores when considering all three fault models and all three signal, respectively.

*Comparison with random testing* We compare code coverage and mutation score of the synthesized adaptive test strategies and random testing when executed for 0.1k, 1k, 10k, 100k time steps. The suffix “k” multiplies by $10^3$. We use a uniform random distribution for choosing random values for all input signals, where reset is with $10\%$ probability 1, and all other signals are with 50% probability 1.

The coverage and mutation scores are listed in Table 5. Coverage was measured with gcov. The table is built as follows: the different testing approaches are shown in the columns. The columns **R**(0.1**k**), **R**(1**k**), **R**(10**k**), **R**(100**k**) refer to random testing with increasing numbers of input stimuli, and the columns **S**(80) and **S**(80) + **R**(10**k**) refer to the synthesized test strategies and the test strategies in combination with **R**(10**k**).

Overall, random testing achieves high code and mutation scores when executed on the source code of the FDIR component, but can only be used if a concrete implementation of the system is available. The adaptive test strategies, on the other hand, are directly derived from the specification and independent from a concrete implementation. They can be used to derive tests if the system is still under development. Parts of the implementation which refine the specification, or which are not specified at all are not necessarily covered. The last column **S**(80) + **R**(10**k**) also shows that the synthesized test strategies improve coverage and mutation scores over **R**(10**k**). Moreover, the test strategies are able to kill three mutants that are missed by all random test sequences. These mutants can only be killed when executing certain input/output sequences and it is very unlikely for random testing to hit one of them.

**Table 5:** Overview of coverage and mutation score by testing approach

Metric	Random stimuli				Adapt. strategies	
	**R**(0.1**k**)	**R**(1**k)**	**R**(10**k)**	**R**(100**k)**	**S**(80)	**S**(80) + **R**(10**k)**
	(%)	(%)	(%)	(%)	(%)	(%)
Line	91.5	95.7	96.8	100.0	83.0	97.9
Branch	85.4	89.6	89.6	93.8	70.8	91.7
Mutation	88.0	92.8	94.0	98.0	78.3	97.6

## Conclusion

We have presented a new approach to compute adaptive test strategies from temporal logic specifications using reactive synthesis with partial information. The computed test strategies reveal all instances of a user-defined fault class for every realization of a given specification. Thus, they do not rely on implementation details, which is important for products that are still under development or for standards that will be implemented by multiple vendors. Our approach is sound but incomplete in general, i.e., may fail to find test strategies even if they exist. However, for many interesting cases, we showed that it is both sound and complete.

The worst-case complexity is doubly exponential in the specification size, but in our setting, the specifications are typically small. This also makes our approach an interesting application for reactive synthesis. Our experiments demonstrate that our approach can compute meaningful tests for specifications of industrial size and that the computed strategies are capable of detecting faults hidden in paths that are unlikely to be activated by random input sequences.

We have applied our approach in a case study to the fault detection, isolation and recovery component of the satellite Eu:CROPIS . The computed test suite, based only on three different types of faults, achieves a line coverage, branch coverage, and mutation score of 83.0%, 70.8%, ad 78.3%, respectively, relying on information solely available from the specification. The approach also allows us to detect faults that require complex input sequences and are unlikely detected by using random testing.

Current directions for future work include improving scalability, success-rate, and usability of our approach. To this end, we are investigating using random testing for inputs in the strategies that are not fixed to single values, and best-effort strategies [19, 20] for the case that there are no test strategies that can guarantee triggering the fault. Another direction for future work is research on evaluating LTL properties specified on infinite paths on finite traces to improve the evaluation process when executing the derived strategies.

## Footnotes

1 While the semantics of LTL are defined over infinite execution traces, we can only run the tests for a finite amount of time. This can result in inconclusive verdicts [6]. We exclude this issue from the scope of this paper, relying on the user to judge when tests have been executed long enough, and on existing research on interpreting LTL over finite traces [14, 15, 27, 39].

2 PARTYStrategy, https://www.iaik.tugraz.at/content/research/scos/tools/.

3 This fault model is different from the standard fault model in mutation testing, which considers simple faults in a concrete implementation that can affect multiple outputs.

4 The word “complete” indicates that every considered fault is revealed at every output. The word “universal” indicates that this is achieved for every (otherwise correct) system.

5 This is (at least partially) confirmed by our test strategy synthesis tool: it reports that no test strategy with less than 12 states can satisfy Eq. 6.

6 PARTYStrategy, https://www.iaik.tugraz.at/content/research/scos/tools/.

7 Given that the user has decided that we have waited long enough for $\mathtt{safemode}\,$ to become true.

## References

[1] Acree AT, Budd TA, DeMillo RA, Lipton RJ, Sayward FG (1979) Mutation analysis. Technical report GIT-ICS-79/08, Georgia Institute of Technology, Atlanta, Georgia

[2] Aichernig BK, Brandl H, Jöbstl E, Krenn W, Schlick R (2015). Killing strategies for model-based mutation testing. Softw Test Verif Reliab.

[3] Alur R, Courcoubetis C, Yannakakis M (1995) Distinguishing tests for nondeterministic and probabilistic machines. In: Leighton FT, Borodin A (eds) Proceedings of the twenty-seventh annual ACM symposium on theory of computing, 29 May–1 June 1995, Las Vegas, Nevada, USA. ACM, pp 363–372

[4] Ammann P, Ding W, Xu D (2001) Using a model checker to test safety properties. In: 7th International conference on engineering of complex computer systems (ICECCS 2001), 11–13 June 2001. Sweden. IEEE Computer Society, Skövde, pp 212–221

[5] Armoni Roy, Fix Limor, Flaisher Alon, Grumberg Orna, Piterman Nir, Tiemeyer Andreas, Vardi Moshe Y. (2003). Enhanced Vacuity Detection in Linear Temporal Logic. Computer Aided Verification.

[6] Bauer A, Leucker M, Schallhart C (2011). Runtime verification for LTL and TLTL. ACM Trans Softw Eng Methodol.

[7] Beer I, Ben-David S, Eisner C, Rodeh Y (2001). Efficient detection of vacuity in temporal model checking. Formal Methods Syst Des.

[8] Blass A, Gurevich Y, Nachmanson L, Veanes M Play to test. In: Grieskamp and Weise [26], pp 32–46

[9] Bloem R, Chatterjee K, Jobstmann B, Clarke EM, Henzinger TA, Veith H, Bloem R (2018). Graph games and reactive synthesis. Handbook of model checking.

[10] Bloem R, Könighofer R, Pill I, Röck F (2016) Synthesizing adaptive test strategies from temporal logic specifications. In: Piskac R, Talupur M (eds) 2016 Formal methods in computer-aided design, FMCAD 2016, Mountain View, CA, USA, 3–6 Oct 2016. IEEE, pp 17–24 10.1007/s10703-019-00338-9 PMC6892341 31866704

[11] Boroday S, Petrenko A, Groz R (2007). Can a model checker generate tests for non-deterministic systems?. Electr Notes Theor Comput Sci.

[12] Clarke EM, Emerson EA, Kozen D (1981). Design and synthesis of synchronization skeletons using branching-time temporal logic. Logics of programs, workshop, Yorktown Heights, New York, USA, May 1981, volume 131 of lecture notes in computer science.

[13] David A, Larsen KG, Li S, Nielsen B (2008) A game-theoretic approach to real-time system testing. In: Sciuto D (ed) Design, automation and test in Europe, DATE 2008, Munich, Germany, March 10–14, 2008. ACM, pp 486–491

[14] De Giacomo G, De Masellis R, Montali M (2014) Reasoning on LTL on finite traces: Insensitivity to infiniteness. In: Brodley CE, Stone P (eds) Proceedings of the twenty-eighth AAAI conference on artificial intelligence, July 27–31, 2014, Québec City, Québec, Canada. AAAI Press, pp 1027–1033

[15] De Giacomo G, Vardi MY (2013) Linear temporal logic and linear dynamic logic on finite traces. In: Rossi F (ed) IJCAI 2013, Proceedings of the 23rd international joint conference on artificial intelligence, Beijing, China, August 3–9, 2013. IJCAI/AAAI, pp 854–860

[16] DeMillo RA, Lipton RJ, Sayward FG (1978). Hints on test data selection: help for the practicing programmer. IEEE Comput.

[17] Dillig Isil, Dillig Thomas, McMillan Kenneth L., Aiken Alex (2012). Minimum Satisfying Assignments for SMT. Computer Aided Verification.

[18] Ehlers R (2012). Symbolic bounded synthesis. Form Methods Syst Des.

[19] Faella M (2008) Best-effort strategies for losing states. CoRR arXiv:0811.1664

[20] Faella M (2009) Admissible strategies in infinite games over graphs. In: Královic R, Niwinski D (ed) Proceedings of the 34th international symposium on mathematical foundations of computer science 2009, MFCS 2009, Novy Smokovec, High Tatras, Slovakia, August 24–28, 2009. Volume 5734 of lecture notes in computer science. Springer, pp 307–318

[21] Finkbeiner B, Schewe S (2013). Bounded synthesis. STTT.

[22] Fraser G, Ammann P (2008) Reachability and propagation for LTL requirements testing. In: Zhu H (ed) Proceedings of the eighth international conference on quality software, QSIC 2008, 12–13 August 2008, Oxford, UK. IEEE Computer Society, pp 189–198

[23] Fraser G, Wotawa F (2007) Test-case generation and coverage analysis for nondeterministic systems using model-checkers. In: Proceedings of the second international conference on software engineering advances (ICSEA 2007), August 25–31, 2007, Cap Esterel, French Riviera, France. IEEE Computer Society, p 45

[24] Fraser G, Wotawa F, Ammann P (2009). Issues in using model checkers for test case generation. J Syst Softw.

[25] Fraser G, Wotawa F, Ammann P (2009). Testing with model checkers: a survey. Softw Test Verif Reliab.

[26] Grieskamp W, Weise C (eds) (2006) Formal approaches to software testing, 5th international workshop, FATES 2005, Edinburgh, UK, July 11, 2005, revised selected papers, vol 3997. Lecture notes in computer science. Springer

[27] Havelund K, Rosu G (2001) Monitoring programs using rewriting. In: 16th IEEE international conference on automated software engineering (ASE 2001), 26–29 November 2001, Coronado Island, San Diego, CA, USA. IEEE Computer Society, pp 135–143

[28] Hierons RM (2006). Applying adaptive test cases to nondeterministic implementations. Inf Process Lett.

[29] Jia Y, Harman M (2011). An analysis and survey of the development of mutation testing. IEEE Trans Softw Eng.

[30] Jin HS, Ravi K, Somenzi F (2004). Fate and free will in error traces. STTT.

[31] Khalimov A, Jacobs S, Bloem R (2013) PARTY parameterized synthesis of token rings. In: Sharygina N, Veith H (eds) Proceedings of the 25th international conference on computer aided verification—CAV 2013, Saint Petersburg, Russia, July 13–19, 2013. Volume 8044 of lecture notes in computer science. Springer, pp 928–933

[32] Könighofer R, Hofferek G, Bloem R (2013). Debugging formal specifications: a practical approach using model-based diagnosis and counterstrategies. STTT.

[33] Kupfermant Orna, Vardit Moshe Y. (2000). Synthesis with Incomplete Informatio. Applied Logic Series.

[34] Kupferman O, Vardi MY (2003). Vacuity detection in temporal model checking. STTT.

[35] Luo G, von Bochmann G, Petrenko A (1994). Test selection based on communicating nondeterministic finite-state machines using a generalized wp-method. IEEE Trans Softw Eng.

[36] Martin DA (1975). Borel determinacy. Ann Math.

[37] Mathur AP (2008). Foundations of software testing.

[38] Miyase K, Kajihara S (2004). XID: don’t care identification of test patterns for combinational circuits. IEEE Trans CAD Integr Circuits Syst.

[39] Morgenstern A, Gesell M, Schneider K (2012) An asymptotically correct finite path semantics for LTL. In: Bjørner N, Voronkov A (eds) Proceedings of the 18th international conference on logic for programming, artificial intelligence, and reasoning, LPAR-18, Mérida, Venezuela, March 11–15, 2012. Volume 7180 of lecture notes in computer science. Springer, pp 304–319

[40] Nachmanson L, Veanes M, Schulte W, Tillmann N, Grieskamp W (2004) Optimal strategies for testing nondeterministic systems. In: Avrunin GS, Rothermel G (eds) Proceedings of the ACM/SIGSOFT international symposium on software testing and analysis, ISSTA 2004, Boston, MA, USA, July 11–14, 2004. ACM, pp 55–64

[41] Offutt AJ (1992). Investigations of the software testing coupling effect. ACM Trans Softw Eng Methodol.

[42] Petrenko A, da Silva Simão A, Yevtushenko N (2012) Generating checking sequences for nondeterministic finite state machines. In: Antoniol G, Bertolino A, Labiche Y (eds) Fifth IEEE international conference on software testing, verification and validation, ICST 2012, Montreal, QC, Canada, April 17–21, 2012. IEEE Computer Society, pp 310–319

[43] Petrenko A, Simão A (2015). Generalizing the ds-methods for testing non-deterministic fsms. Comput J.

[44] Petrenko A, Yevtushenko N. Conformance tests as checking experiments for partial nondeterministic FSM. In: Grieskamp and Weise [26], pp 118–133

[45] Petrenko A, Yevtushenko N (2014) Adaptive testing of nondeterministic systems with FSM. In: 15th international IEEE symposium on high-assurance systems engineering, HASE 2014, Miami Beach, FL, USA, January 9–11, 2014. IEEE Computer Society, pp 224–228

[46] Pnueli A (1977) The temporal logic of programs. In: 18th annual symposium on foundations of computer science, Providence, Rhode Island, USA, 31 October–1 November 1977. IEEE Computer Society, pp 46–57

[47] Pnueli A, Rosner R (1989) On the synthesis of a reactive module. In: Conference record of the sixteenth annual ACM symposium on principles of programming languages, Austin, Texas, USA, January 11–13, 1989. ACM Press, pp 179–190

[48] Queille J. P., Sifakis J. (1982). Specification and verification of concurrent systems in CESAR. Lecture Notes in Computer Science.

[49] Tretmans J (1996). Conformance testing with labelled transition systems: implementation relations and test generation. Comput Netw ISDN Syst.

[50] Tan L, Sokolsky O, Lee I (2004) Specification-based testing with linear temporal logic. In: Zhang D, Grégoire É, DeGroot D (eds) Proceedings of the 2004 IEEE international conference on information reuse and integration, IRI—2004, November 8–10, 2004, Las Vegas Hilton, Las Vegas, NV, USA. IEEE Systems, Man, and Cybernetics Society, pp 493–498

[51] Tipaldi M, Bruenjes B (2015). Survey on fault detection, isolation, and recovery strategies in the space domain. J Aerosp Inf Syst.

[52] Yannakakis M (2004) Testing, optimizaton, and games. In: Díaz J, Karhumäki J, Lepistö A, Sannella D (eds) Proceedings of the automata, languages and programming: 31st international colloquium, ICALP 2004, Turku, Finland, July 12–16, 2004. Volume 3142 of lecture notes in computer science. Springer, pp 28–45

